# Molecular mechanisms, pathogenesis and therapeutic advances of *Candida albicans* in oral candidiasis

**DOI:** 10.3389/fcimb.2026.1918424

**Published:** 2026-09-15

**Authors:** Binaya Krushna Sahu, Priyanka Debta, Sudipta Kumar Patra, Mahesh Chandra Sahu, Tapan Kumar Mohanta, Sujogya Kumar Panda

**Affiliations:** 1 Centre for Biotechnology, Siksha ‘O’ Anusandhan (Deemed to be University), Bhubaneswar, Odisha, India; 2 Department of Oral Pathology and Microbiology, Institution of Dental Sciences, Siksha ‘O’ Anusandhan (Deemed to be University), Bhubaneswar, Odisha, India; 3 Department of Orthopedics, Kalinga Institute of Medical Sciences, Bhubaneswar, Odisha, India; 4 Microbiology Division, Indian Council of Medical Research (ICMR)-Regional Medical Research Centre, Bhubaneswar, Odisha, India; 5 Department of Genetic Research, Institute for Research and Medical Consultations, Imam Abdulrahman Bin Faisal University, Dammam, Saudi Arabia

**Keywords:** antifungal resistance, biofilm, *Candida albicans*, host-directed therapy, morphogenesis, photodynamic therapy, probiotics

## Abstract

Oral candidiasis (OC), caused mainly by *Candida albicans*, is a common opportunistic fungal infection and typically affects immunocompromised individuals. The rise in antifungal resistance, biofilm-associated persistence, and immune evasion mechanisms has complicated OC management. This review examines in depth the molecular pathogenesis of *C. albicans* in OC, including virulence factors associated with adhesion, morphogenesis, quorum sensing, immune evasion, and biofilm formation. The review highlights the central roles of the cAMP-PKA, MAPK, Rim101, and TOR signaling pathways in regulating yeast-to-hyphal transition, virulence, stress adaptation, and biofilm development. In particular, dysregulation of these pathways promotes filamentation and biofilm maturation, contributing to persistent infection and reduced antifungal susceptibility. Conventional antifungal therapies, including azoles, polyenes, and echinocandins, remain important for OC management; however, biofilm-associated tolerance and emerging resistance can limit their effectiveness, emphasizing the need for complementary therapeutic approaches. Emerging strategies, including host-directed therapies, probiotics, photodynamic therapy (PDT), phytotherapeutics, essential oils, phenolic compounds, nanoparticles, and antimicrobial peptides, show promising antibiofilm and adjunctive potential. Clinical and experimental evidence indicates that PDT can reduce *Candida* burden, while probiotics may reduce oral *Candida* colonization and interfere with biofilm development. Furthermore, natural products, nanoparticles, and antimicrobial peptides may enhance conventional antifungal activity through complementary mechanisms. The reviewed evidence suggests that targeting fungal virulence and biofilm-associated mechanisms alongside conventional antifungal therapy may provide a more effective strategy for recurrent and drug-resistant OC. Future research should prioritize standardized clinical evaluation of antibiofilm therapies, microbiome-directed interventions, personalized treatment approaches, and combination therapies to improve therapeutic outcomes.

## Introduction

Oral candidiasis (OC), primarily caused by the opportunistic fungal pathogen *Candida* (*C.*) *albicans*, is a common fungal infection of the mucosal surfaces of the oral cavity (Lenka et al., 2020; Vila et al., 2020). OC predominantly affects numerous populations, particularly immunocompromised patients with HIV/AIDS, cancer patients and those receiving immunosuppressive therapies and medications (Egusa et al., 2008; Mallick et al., 2018; Patel, 2022; Rath et al., 2018; Singh and Kumari, 2024). OC may cause mild to severe infections, leading to discomfort and difficulty swallowing; in severely immunocompromised individuals, it may occasionally progress to systemic dissemination (Debta et al., 2020; Vila et al., 2020). The pathogenesis of *C. albicans* in OC is a complex process driven by interactions between the host and the pathogen. It is further influenced by host immunity, the presence of microbial flora, and other environmental factors (Hebecker et al., 2014; Williams and Lewis, 2011). New insights into host-microbe interactions have deepened our understanding of the fundamental mechanisms underlying *Candida* pathogenicity. Adhesion to host cells, biofilm formation, immune evasion, morphogenesis, and quorum sensing are major factors required for the establishment and maintenance of infection (Atriwal et al., 2021; Bojang et al., 2021). The transition of *C. albicans* from a commensal to a pathogenic organism is tightly regulated by environmental conditions and host immune responses, making it a highly adaptable pathogen (Chow et al., 2021; Sudbery, 2011).

The development of antifungal agents for *Candida* species has advanced significantly over the last few decades, largely due to the increasing incidence of candidiasis and the emergence of antifungal resistance. Although several effective antifungal therapies are available, there remains a need for new and improved therapeutic options because of challenges associated with antifungal resistance, disease recurrence, biofilm-associated antifungal tolerance, and impaired host immune responses. Recent therapeutic advances in the management of OC include novel antifungal agents, host-directed therapies, probiotics, photodynamic therapy, and other complementary approaches (Pfaller et al., 2019; Rodrigues et al., 2019; Sharma, 2019). **Figure 1** summarizes the principal cellular targets of currently used antifungal agents against *Candida* species and illustrates key mechanisms contributing to antifungal resistance. Polyenes, such as amphotericin B and nystatin, interact with membrane ergosterol and disrupt plasma membrane integrity; azoles inhibit ergosterol biosynthesis primarily through targeting Erg11; echinocandins inhibit β-(1,3)-glucan synthesis through the Fks glucan synthase complex; and nucleoside analogues such as flucytosine interfere with fungal nucleic acid synthesis. The resistance mechanisms, including alterations in *ERG11* and increased expression of drug-efflux transporters, can reduce antifungal susceptibility and contribute to therapeutic failure.

**Figure 1:**
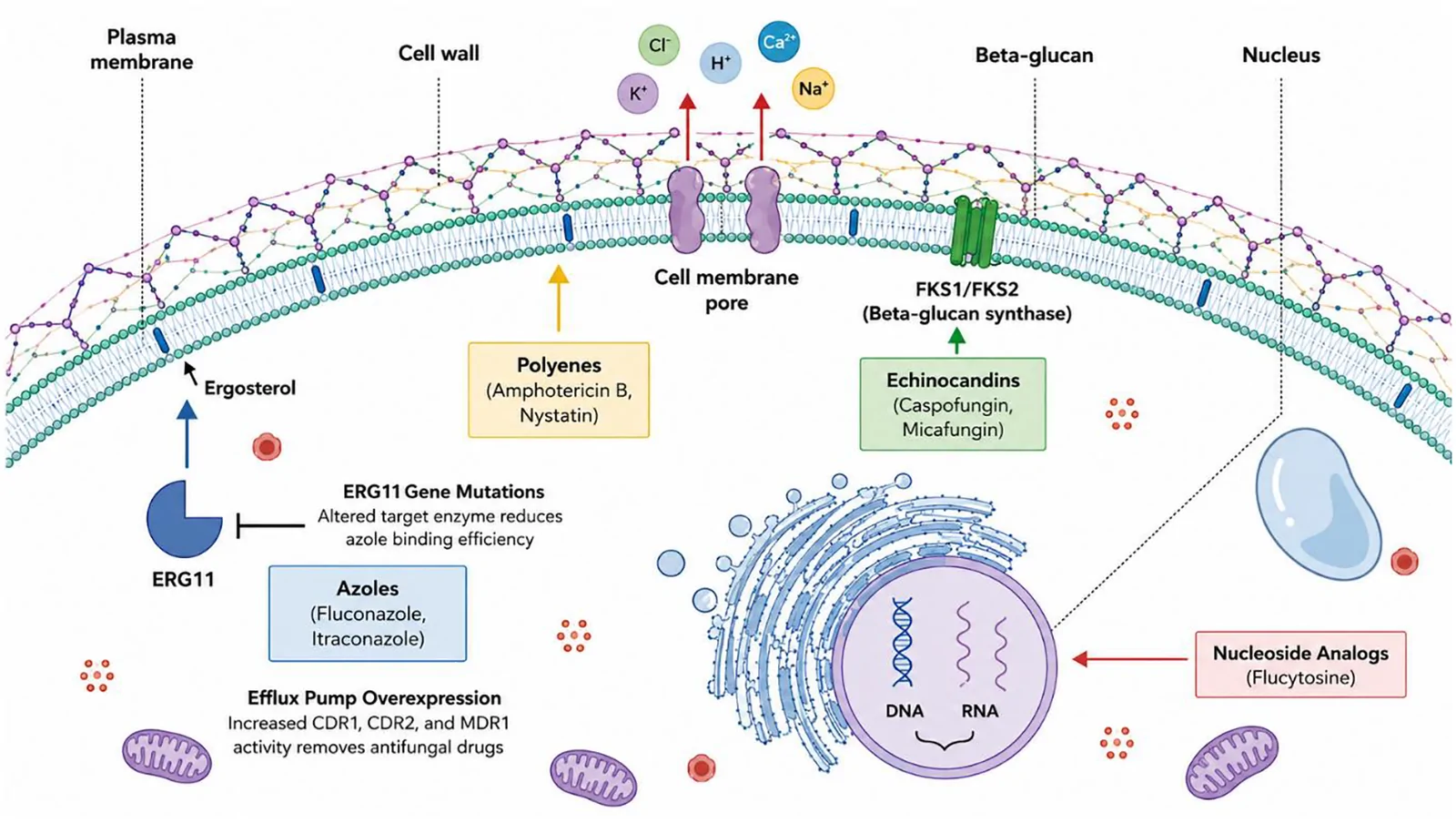
The main targets of antifungal drugs and their resistance mechanisms in *Candida albicans*. Polyene antifungal agents induce polymerization, forming pores in the fungal plasma membrane by interacting with ergosterol. Azole antifungal agents target ergosterol biosynthesis. Echinocandin antifungal agents inhibit β-(1,3)-glucan synthase (*FKS1/FKS2*), leading to disruption of yeast cell wall synthesis. Nucleoside analogs inhibit fungal DNA and RNA synthesis by disrupting nucleotide metabolism. Common resistance mechanisms in *C. albicans*, such as mutations in the *ERG11* gene, overexpression of efflux pump genes (*CDR1, CDR2*, and *MDR1*), and biofilm production, all of which decrease *C. albicans* susceptibility to antifungals and increase its survival. Adapted from Czajka et al. (2023).

This review will summarize the molecular mechanisms underlying *C. albicans* pathogenesi*s* in OC and will consider recent therapeutic advances in this disease. An informed understanding of these mechanisms could enable the development of targeted interventions aimed at preventing fungal colonization, overcoming antifungal resistance and biofilm-associated tolerance, and improving treatment outcomes, particularly in individuals with chronic, recurrent, or treatment-refractory OC.

## Adhesion and biofilm formation in *Candida* pathogenesis

The capacity of *Candida* species to adhere to host surfaces and form biofilms is usually considered the most important factor in their pathogenicity in OC (Arzmi et al., 2014). The adhesion process is more pertinent in the oral cavity, the gastrointestinal tract, and on mucosal surfaces. Adhesion is an important factor in colonization because *C. albicans* must establish a strong attachment to epithelial cells despite turnover and host defense mechanisms. The attachment is enhanced by fungal adhesins, including agglutinin-like sequence (Als) proteins (Als3 and Als1), hyphal wall protein 1 (Hwp1), and epithelial adhesin (Eap1), which recognize and bind to host-cell ligands or extracellular matrix components. These adhesins enable both yeast and hyphal forms of the fungus to attach not only to epithelial cells but also to inert surfaces such as dental prosthetics, catheters, and other medical devices, contributing to both mucosal and systemic infections (Modrzewska and Kurnatowski, 2015; Sudbery, 2011). Als3 mediates the attachment of fungal cells to host proteins, such as E-cadherin and N-cadherin, on epithelial cells. It promotes endocytosis by host cells, while Als1 contributes to both cell-cell adhesion and biofilm formation, thereby enhancing fungal colonization and persistence. Hwp1 acts as a substrate for host transglutaminase, forming covalent bonds with epithelial cells, stabilizing adhesion, and initiating biofilm formation in the oral cavity and esophagus. Eap1 is a glycosylphosphatidylinositol (GPI)-anchored cell wall protein in *C. albicans* that plays a critical role in adhesion, a major virulence factor. Eap1 facilitates the initial attachment of fungal cells to host surfaces, including epithelial cells and abiotic surfaces such as medical devices, and plays a critical role in early biofilm development (de Groot et al., 2013; Hoyer et al., 2008; Klis et al., 2009; Modrzewska and Kurnatowski, 2015). Ssa1 (Stress-seventy subfamily A protein 1) is a member of the Hsp70 family that also functions as an invasin. It binds to the host-cell receptors and facilitates fungal entry into epithelial and endothelial cells. Beyond these specific adhesins, cell-surface hydrophobicity also influences adhesion. It enhances the attachment of fungal cells to hydrophobic host surfaces, such as the oral mucosa (Arzmi et al., 2014).

Additionally, *C. albicans* secretes aspartyl proteinases (Saps), encoded by at least 10 genes (*SAP1–SAP10*), that degrade host extracellular matrix components, expose binding sites, and facilitate stronger attachment. Biofilms, a haracteristic feature of *Candida* infections, are facilitated by fungal adhesins composed of β-1,3-glucan, β-1,6-glucan, mannans, chitin, and proteins (Tsui et al., 2016). The ECM is primarily composed of glycoproteins (55%), carbohydrates (25%), lipids (15%), and nucleic acids (5%). Glycoproteins include adhesins and regulatory proteins such as Als1, Als3, Hwp1, Eap1, Pga1, and others. Carbohydrates consist mainly of β-glucans, mannans, and chitin oligosaccharides. Lipids are predominantly glycerolipids (99.5%), with minor contributions from sphingolipids and glycolipids. Extracellular DNA (eDNA) is the nucleic acid fraction that contributes to structural integrity and immune evasion (**Figure 2**). This self-produced extracellular matrix provides physical protection against antifungal agents and shields the fungal cells from host immune responses (Nett et al., 2010; Ramage et al., 2005). Biofilm-associated cells exhibit significantly enhanced resistance to antifungal drugs, often up to 1,000-fold greater than that of their planktonic counterparts (Arzmi et al., 2014; Fanning and Mitchell, 2012; Rao et al., 2021).

**Figure 2:**
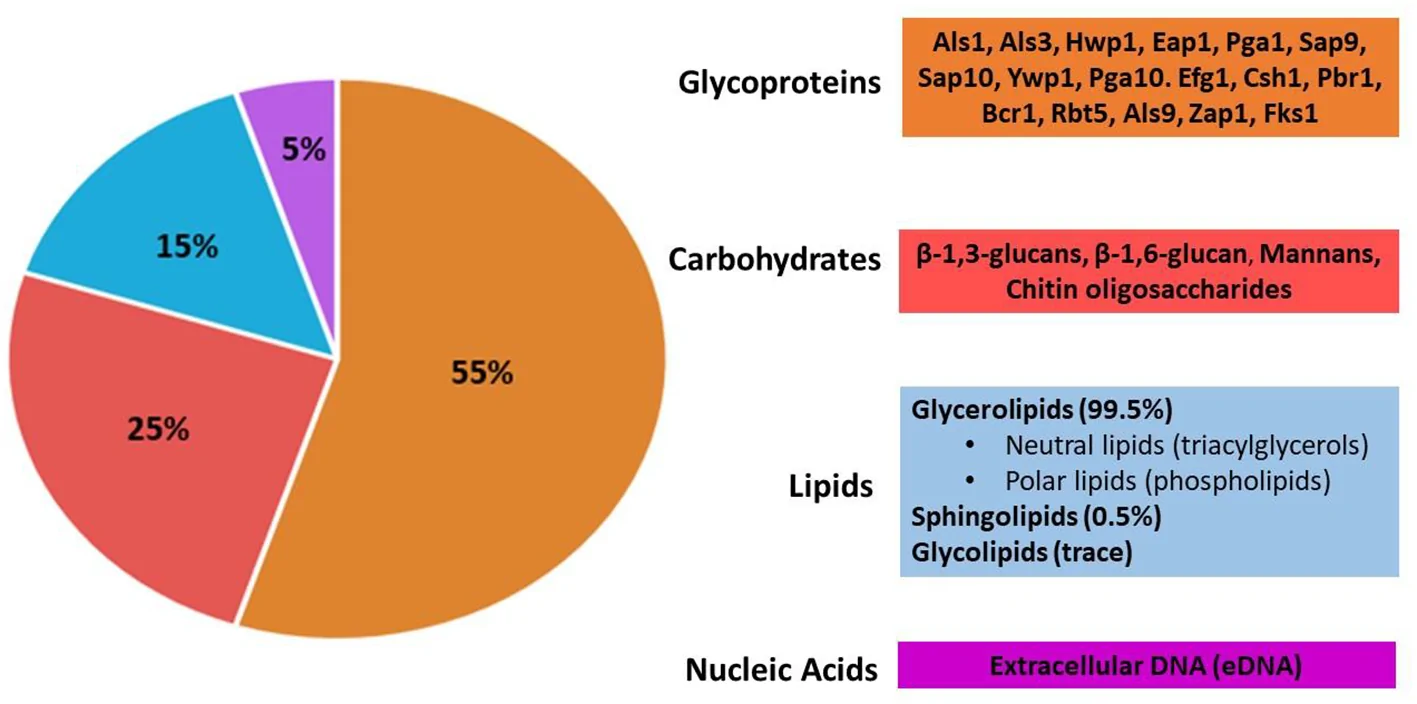
Composition of the extracellular matrix in *C. albicans* biofilm.

Biofilm formation occurs in three distinct stages: initial adhesion, proliferation, and maturation. During the maturation phase, the secretion of extracellular polymeric substances (EPS) is upregulated, forming a protective scaffold that supports nutrient exchange and waste removal (Ramage et al., 2005; Seneviratne et al., 2008). During biofilm formation, *C. albicans* transitions between yeast and hyphal forms, which are regulated by morphogenetic genes such as Efg1 and Cph1 (Huang, 2012; Kumar and Kumar, 2024). Hyphal cells penetrate the host tissues, enhancing colonization and structural stability. Additionally, quorum-sensing molecules (farnesol) regulate cell morphology and biofilm architecture, balancing hyphal and yeast forms to optimize biofilm structure (Polke et al., 2018; Tian et al., 2021). Biofilm formation not only facilitates persistent infections but also allows *Candida* to colonize diverse niches within the host (**Table 1**). This interaction disrupts the immune response, leading to chronic inflammation (Mayer et al., 2013). Such biofilms are frequently implicated in infections associated with medical devices, further highlighting their clinical relevance (Lazãr and Chifiriuc, 2010; Sudbery, 2011).

**Table 1:** Stages and functions of *C. albicans* pathogenesis and biofilm development.

Stage	Function
Initial adhesion	Yeast cells adhere to epithelial cells via adhesins such as Als3, Hwp1, and Eap1, thereby anchoring the fungus to the oral mucosa and initiating colonization.
Germ tube formation	In response to environmental signals (temperature, pH), yeast cells form germ tubes- early hyphal projections that signify morphological switching.
Hyphal transition	Hyphae penetrate deeper tissues, a process regulated by pathways such as cAMP-PKA and transcription factors (Efg1), thereby enhancing tissue invasion and immune evasion.
Biofilm maturation	Biofilm develops into a dense, protective matrix (β-glucans, mannans) containing yeast, hyphae, and ECM, thereby increasing antifungal resistance and shielding against immune responses.
Host cell invasion	Hyphae, through proteins such as Als3 and toxins such as candidalysin, breach epithelial barriers, inducing host cell damage and inflammation.
Immune evasion	*C. albicans* masks β-glucans with mannoproteins and secretes Saps to degrade host defense proteins, preventing recognition and phagocytosis.
Persistent infection	Chronic colonization is established by biofilm resilience, immunosuppression, and fungal adaptability, especially in immunocompromised hosts.

## Morphogenesis in *C. albicans* pathogenesis

The virulence traits of *C. albicans* are its ability to undergo morphogenesis, a reversible transition between yeast, pseudohyphal, and hyphal forms. This morphological flexibility is crucial for tissue invasion, immune evasion, and biofilm formation (**Figure 3**). The yeast-to-hypha transition is tightly regulated by environmental signals, such as pH, carbon sources, temperature, and nutrient availability, which activate specific signaling pathways and transcription factors (Biswas et al., 2007; Chow et al., 2021; Debta et al., 2020; Mayer et al., 2013).

**Figure 3:**
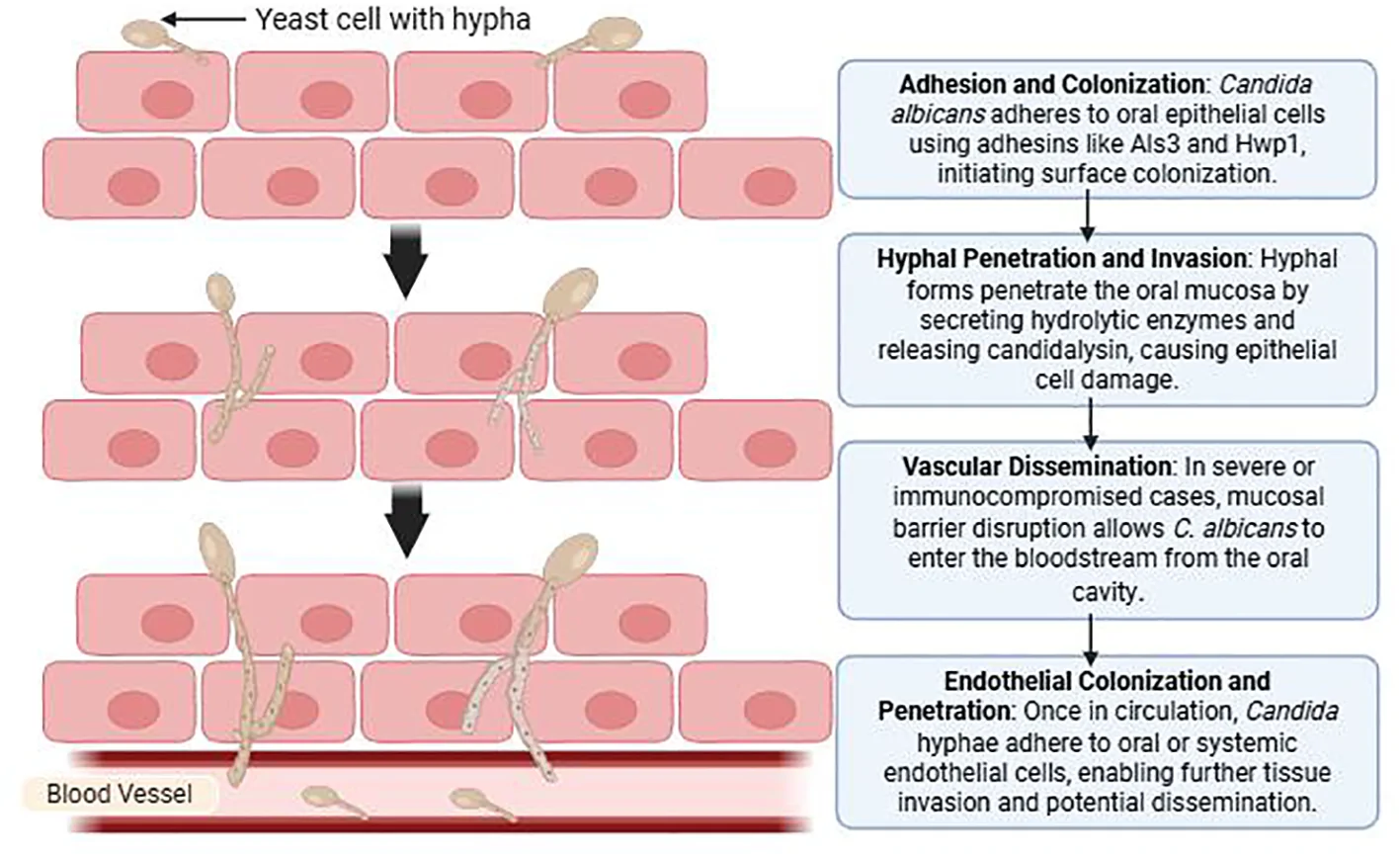
Successive stages of *C. albicans* invasion in oral candidiasis.

## Modulation of *C. albicans* morphogenesis by the cAMP-PKA pathway

In *C. albicans*, the cyclic AMP protein kinase A (cAMP-PKA) signaling cascade is the main regulatory pathway of morphogenesis and virulence. The specificity of environmental signals is processed to drive morphological changes, particularly the transition from yeast-to-hypha transition. Surface receptors and G-protein-coupled signaling pathways detect environmental signals, including temperature, CO_2_ concentration, pH, and nutrients (Braunsdorf et al., 2016). The G-protein Gpa2 interacts with the membrane-associated adenylyl cyclase Cyr1 (also called Cdc35) to activate the pathway. *CYR1* circularly encodes Cyr1 and converts ATP to cAMP in response to upstream signals (Hall et al., 2009; McDonough and Rodriguez, 2012). The cAMP-PKA pathway is critical because it links environmental detection (via signal classification) to downstream signaling. Once upstream signals activate Cyr1, it generates cAMP, which activates protein kinase A (PKA), a principal regulator of the morphogenetic transition in *C. albicans* (Hogan and Muhlschlegel, 2011; Hogan and Sundstrom, 2009). The cAMP-PKA signaling pathway regulates the fungal cell’s ability to adapt to host environments and influences the expression of key hyphal-specific genes, such as *HWP1* and *ALS3*. In *C. albicans*, the endocellular eukaryotic enzyme PKA (protein kinase A) is a heterotetramer composed of two regulatory Bcy1 subunits (which discriminate between cAMP and cGMP) and two catalytic Tpk1 and Tpk2 subunits (Hall et al., 2009; Hogan and Sundstrom, 2009; McDonough and Rodriguez, 2012; Sudbery, 2011). The *BCY1* gene encodes the regulatory Bcy1 subunits, which sense cAMP. The regulatory subunit binds cAMP in response to environmental signals, such as thermal, nutrient, or pH signals. When cAMP binds Bcy1, it elicits a conformational change in the Bcy1 regulatory subunits, which causes the dissociation of the Tpk1 and Tpk2 catalytic subunits from the regulatory complex and activation of PKA. Once released from the regulatory subunits, Tpk1 and Tpk2, encoded by *TPK1* and *TPK2*, respectively, can phosphorylate downstream targets. A cellular response occurs in the cell due to PKA phosphorylation, most notably in transcriptional regulation and changes in molecular morphology (Grahl et al., 2015; Hall et al., 2009; Liu, 2001; McDonough and Rodriguez, 2012).

## Phosphorylation of transcription factors in the cAMP-PKA pathway

When the protein kinase A pathway is activated by cAMP, a phosphorylation cascade occurs, including phosphorylation of important transcription factors. The transcription factors modified by PKA include Efg1 (enhanced filamentous growth protein 1), which is crucial to regulating hyphal development and morphogenesis in *C. albicans* (Hall et al., 2009; Hogan and Sundstrom, 2009; Sudbery, 2011).

The *EFG1* gene encodes the Efg1 protein, a transcription factor that acts as a master regulator of filamentous growth in *C. albicans*. This protein is central to the transition from yeast to hyphal growth, a process critical for the fungus’s virulence. In its unphosphorylated state, Efg1 is inactive and bound by repressive complexes that prevent the expression of hyphal-specific genes. However, when environmental signals activate the cAMP-PKA pathway, the catalytic subunits of PKA (Tpk1 and Tpk2) phosphorylate Efg1, thereby activating it and promoting its dissociation from repressive complexes. Once Efg1 is phosphorylated, it acts as a transcriptional activator, promoting the expression of several genes essential for hyphal formation (Hall et al., 2009; Liu, 2001). Three of the most important genes activated by Efg1 are:

### HWP1

*HWP1* encodes a hypha-associated cell wall protein that plays a vital role in the adhesion of *C. albicans* hyphae to host tissues. As the fungus transitions to the hyphal form, *HWP1* expression is upregulated by Efg1, facilitating stable attachment to host surfaces. The Hwp1 protein, which is covalently bound to the N-terminal domain, serves as a substrate for host transglutaminases, rendering it resistant to removal by washing and making it an essential factor in both initial adhesion and the maintenance of fungal growth in tissues. Its role in stabilizing fungal attachment to host surfaces contributes significantly to biofilm formation and the persistence of chronic infection (Klis et al., 2009; Modrzewska and Kurnatowski, 2015).

### ALS3

*ALS3* encodes an adhesin protein essential for *C. albicans* interaction with host epithelial cells. The *ALS3* protein binds to host cadherins, enabling *Candida* cells to invade host tissue and thereby promoting colonization and pathogenesis. Therefore, by biologically facilitating *C. albicans*’s adherence and invasion of cells, *ALS3* enables biofilm formation. Biofilm formation is a key factor in *C. albicans*’s ability to develop persistent infections, especially in mucosal tissues such as the oral cavity and genital tract. Left untreated, biofilm infections are resistant to treatment and can last months or years. Because somatic cell adhesion cannot occur without prior penetration of host cells by hyphal structures, Efg1 was shown to regulate *ALS3* gene expression. This highlights an important link between hyphal transition and improved adhesion and tissue penetration. In fact, Als3 remains inactive until germ tubes form. As a result, both adhesion and invasion are only possible after or concomitant with hyphal growth (Hoyer et al., 2008; Liu, 2001; Modrzewska and Kurnatowski, 2015).

### ECE1

*ECE1* encodes a protein involved in the synthesis of candidalysin, a peptide toxin that has a direct role in host tissue invasion and immune modulation. Candidalysin disrupts host cell membranes, resulting in cell damage and inflammation that may create an environment for fungal invasion by degrading host physical barriers. Additionally, candidalysin may modulate the immune response by activating host immune responses that, in some cases, allow *C. albicans* to evade immune detection and cause a persistent infection. The expression of *ECE1* is regulated by various factors, including Efg1, and is important for the pathogenesis of *C. albicans*, particularly in mucosal infections (Hu et al., 2023; Naglik et al., 2019; Song et al., 2022).

## Feedback regulation in the cAMP-PKA pathway

The pathway is tightly regulated by feedback inhibition to avoid excessive hyphal growth. Many mechanisms and regulatory proteins control the cAMP-PKA pathway, ensuring that the yeast-to-hyphal transition occurs only under permissive conditions and is effectively inhibited when necessary. Ras1 is a small GTPase that is encoded by the *RAS1* gene. It directly regulates the cAMP-PKA pathway. It directly interacts with adenylyl cyclase (Cyr1), the enzyme that synthesizes cyclic AMP (cAMP) in response to environmental signals. Active Ras1 stimulates Cyr1 (adenylate cyclase), which synthesizes more cAMP, activating the cAMP-PKA pathway. Nutritional sensing and environmental conditions tightly regulate Ras1 activity. In the presence of nutrients, Ras1 is activated, producing cAMP and triggering hyphal growth. In unfavorable conditions (nutrient limitation or stress), Ras1 activity is downregulated to inhibit uncontrolled hyphal growth. Feedback is an essential characteristic that ensures *C. albicans* proceeds to morphogenesis only under favorable conditions that facilitate growth and survival (Grahl et al., 2015; Hall et al., 2009).

In addition to Ras1, two key transcriptional repressors, Nrg1 and Tup1, are involved in the feedback regulation of hyphal-specific gene expression. These proteins inhibit the expression of hyphal-specific genes when environmental conditions are not favorable for filamentation. Nrg1 is a repressor that plays a critical role in maintaining *C. albicans* in the yeast form under certain conditions. In the presence of repressive signals, Nrg1 binds to the promoters of hyphal-specific genes, such as *HWP1* and *ALS3*, preventing their activation. Tup1 is another repressor protein that works alongside Nrg1 to inhibit hyphal growth. It forms complexes with other coregulatory proteins to maintain the yeast form by suppressing hyphal-specific gene expression (Huang, 2012; Liu, 2001; Sudbery, 2011).

For the transition from yeast to hyphae to occur in response to favorable conditions, PKA also phosphorylates the repressors Nrg1 and Tup1. Phosphorylation by PKA prevents the repressive function of these proteins, lifting inhibition of hyphal-specific genes. When PKA phosphorylates Nrg1 and Tup1, the repressors are either inactivated or released from their target genes, allowing transcription of hyphal-specific genes, such as *HWP1* and *ALS3*. This regulatory mechanism allows the fungal cell to transition from a yeast form to a hyphal form very quickly when environmental cues indicate that growth and, eventually, infection are possible. Further, it prevents nonspecific or unregulated hyphal growth in conditions that are not conducive to growth (Dhillon et al., 2003; Liu, 2001; Sudbery, 2011).

## Integration of the cAMP-PKA pathway with other signaling pathways in *C. albicans*

The cAMP-PKA pathway in *C. albicans* does not act in isolation but integrates signals from multiple cellular pathways to regulate morphogenesis, stress responses, and virulence.

### Cph1

The MAPK pathway, especially the Cph1 MAPK cascade, is a critical regulator of morphogenesis, stress response, and virulence in *C. albicans*. Various environmental stresses, including temperature shifts, osmotic pressure, and pH changes, activate the pathway. Cph1 is a transcription factor in the MAPK signaling cascade and can activate or repress transcription of specific target genes critical for filamentation (Dhillon et al., 2003; Huang et al., 2008). The Cph1 MAPK cascade is integrated with the cAMP-PKA pathway to provide a greater amplitude of expression of hyphal-specific genes, since Cph1 promotes *HWP1* and *ALS3* transcription that is required in adhesion and invasion when *C. albicans* grows in the hyphal form. Under stressful conditions, amplification is more pronounced because both the MAPK and cAMP-PKA pathways are required to ensure a sufficient response (Ramírez-Zavala et al., 2013). The cAMP-PKA pathway and the Cph1 MAPK pathway are functionally linked, as both signaling cascades often coordinate and converge on shared downstream targets to regulate fungal development and gene expression, though they operate through distinct intermediate kinase networks. This coordination between these pathways helps ensure that *Candida* hyphal transitions occur only under appropriate environmental conditions for invasion and persistence (Dhillon et al., 2003; Ramírez-Zavala et al., 2013).

### Rim101

The Rim101 pathway is an important signaling cascade that, together with the cAMP-PKA pathway, modulates morphogenesis in *C. albicans*. This pathway helps *C. albicans* sense and respond to environmental pH changes, which are crucial during infection, when it is exposed to varying pH conditions in host tissues (Shimasawa et al., 2023; Yan et al., 2020). Under alkaline pH conditions, as during colonization of mucosal surfaces, the Rim101 pathway drives the expression of hyphal-specific genes. At acidic pH, Rim101 is repressed, preventing the regulation of genes involved in hyphal growth. At an ideal pH, Rim101 cooperates with Efg1 to activate hyphal-specific genes such as HWP1, *ALS3*, and *ECE1* (Kullas et al., 2007; Shimasawa et al., 2023). Rim101 functions in parallel with Efg1 in the cAMP-PKA pathway and is a downstream effector of both the cAMP-PKA and Rim101 pathways. This integration allows *C. albicans* to fine-tune its responses to environmental conditions and, when appropriate, to maximize its morphogenetic switch in response to pH, nutrient availability, and other host-derived signals (Yan et al., 2020).

### TOR pathway

The TOR pathway regulates several cellular activities, including nutrient sensing, cell growth, and stress responses. In *C. albicans*, the TOR pathway links with the cAMP-PKA pathway to help coordinate morphogenesis in response to nutrient acquisition. The TOR pathway monitors nutrient availability in the environment and modulates the cAMP-PKA pathway accordingly. The presence of ample nutrients promotes filamentation and upregulates cAMP-PKA signaling. In contrast, nutrient deprivation promotes TOR inhibition and may also reduce hyphal formation by compromising the cAMP-PKA pathway. Together, the cAMP-PKA and TOR pathways regulate the growth and morphogenesis of *C. albicans*. While TOR regulates cellular metabolism and growth, the cAMP-PKA signaling pathway directly regulates morphological transition, maximizing filamentous growth in the presence of abundant resources and limiting it in the presence of limited resources (Raught et al., 2001; Tong et al., 2021; Weisman, 2016).

## Host-directed therapies in oral candidiasis

The ability of *C. albicans* to evade the host immune response is a major aspect of its pathogenicity and persistence within the host. One important immune-evasion mechanism involves masking cell wall β-glucans, a class of pathogen-associated molecular patterns (PAMPs), readily recognized by host pattern-recognition receptors, such as Dectin-1. In its yeast form, *C. albicans* masks its β-glucans with an outer layer of mannoproteins, preventing immunological recognition and activation of antifungal immune responses, such as phagocytosis and the production of proinflammatory cytokines (Gow et al., 2007). *C. albicans* also uses Saps to degrade host immune proteins, targeting immunoglobulins, complement components, and other host immune defense molecules, inhibiting the host’s ability to mount an effective immune response (Naglik et al., 2003a, 2003b). These enzymes are important in allowing disease by facilitating tissue invasion and neutrophil killing. Another immune evasion strategy is the ability to dynamically remodel its cell wall. In response to immune pressure, *C. albicans* modulates the expression of specific cell wall components, such as chitin and mannan, to evade recognition and enhance resistance to oxidative damage (Komalapriya et al., 2015). This adaptive strategy allows the fungus to persist in environmental extremes within the host, where it can potentially cause damage. In addition, *C. albicans* has a variety of immunomodulatory factors, such as candidalysin, that not only directly harm epithelial cells but can also modulate immune signaling. As candidalysin enables tissue invasion, it can also skew the immune response in ways that promote fungal persistence (Mayer et al., 2013). These strategies will allow *C. albicans* to establish an infection, successfully evade immune defense, and inhabit the niche it has created within the host. Recognizing and understanding these evasion strategies is an invaluable opportunity to create novel therapeutic interventions.

## Quorum sensing in *C. albicans*

Quorum sensing (QS) is an important mechanism for regulating *C. albicans* growth, biofilm, and colonization. QS is the production, release, detection, and response to small signaling molecules, that enable microbial communities to communicate and coordinate behavior in response to environmental context and cell density. In *C. albicans*, farnesol and tyrosol are well-characterized quorum-sensing molecules that regulate many virulence traits, including growth, morphogenesis, and biofilm development, and phenotypic adaptation (Hall et al., 2009; Polke et al., 2018).

Farnesol, a member of the terpenoid family of chemicals, is one of the most studied of these QS molecules in *C. albicans*. Farnesol is produced during yeast growth and negatively regulates hyphal development. At high cell densities, farnesol accumulates and inhibits the yeast-to-hyphal transition, thereby favoring the maintenance of *C. albicans* in the yeast-form state and regulating biofilm development. This density-dependent regulation of morphogenesis may help prevent premature hyphal development and biofilm maturation, allowing biofilm formation to occur under conditions that favor fungal persistence and survival (Han et al., 2011). Farnesol also affects the expression of several biofilm maturation genes and those that confer biofilm resistance to antifungal drugs (Hall et al., 2009). Another phenolic QS molecule, tyrosol, has been implicated in biofilm formation and hyphal growth. Unlike farnesol, tyrosol is known to promote hyphal growth and biofilm formation under certain environmental conditions, such as when *C. albicans* is stressed, and nutrient supply is low (Monteiro et al., 2017). Tyrosol also plays a role in regulating *C. albicans* virulence factors by altering the biofilm matrix composition, thereby enhancing colonization and persistence in the host. Both farnesol and tyrosol are involved in biofilm dispersion, the process by which cells leave the mature biofilm to colonize new locations. Farnesol has been shown to induce cell detachment from the biofilm matrix, promoting the dissemination of *C. albicans* to new sites. At the same time, tyrosol aids this process by modifying the integrity of biofilm structure (Cordeiro et al., 2015; Riekhof and Nickerson, 2017).

## Pathogenesis of oral candidiasis

The development of OC is commonly influenced by systemic risk factors, especially in individuals with an immune-compromised state. Immune suppression is a result of a variety of states, such as HIV/AIDS, a cancer diagnosis, organ transplants, or the use of immunosuppressant drugs (corticosteroids, chemotherapy) (Rodrigues et al., 2019; Sharma, 2019). Immune suppression allows systemic *Candida* species to invade and cause opportunistic infections, including in immunocompromised patients, such as those with diabetes mellitus. This chronic disease allows colonization of mucosal tissue by *C. albicans*. Diseases or health issues that specifically affect endocrine function, including diabetes mellitus, can lead to high blood glucose levels or inappropriate hormonal levels. High glucose levels create a favorable environment for fungal growth, allowing *C. albicans* to colonize mucosal surfaces (Ahmad et al., 2019; Černáková and Rodrigues, 2020; Shahabudin et al., 2024). Other digestive or absorptive disorders, or hormonal hyperactivity, may weaken or impair mucosal defenses, thereby increasing *Candida* colonization. Another human behavior that further increases the risk of OC is prolonged antibiotic use (Jørgensen, 2024; Nikou et al., 2019). Unlike antifungal agents, which specifically target fungal structures and processes, broad-spectrum antibiotics can disrupt the composition of the oral microbiota by reducing susceptible bacterial commensals. This ecological disturbance may reduce microbial competition and facilitate *Candida* overgrowth and persistence on mucosal surfaces (Bondaryk et al., 2013; Hameed et al., 2021; Quindós et al., 2019). Other factors influencing risk include poor nutritional status and other human behaviors such as smoking or heavy drinking that impair the oral microbiome and mucosal integrity. These systemic conditions and human behaviors that increase risk indicate that OC has become a multifactorial risk, requiring a comprehensive approach to care and preventive strategies (Nuncã et al., 2022; Patel, 2022; Pflipsen and Zenchenko, 2017). **Figure 4** summarizes the major systemic and local factors contributing to the pathogenesis of OC. Systemic factors, including immunosuppression, diabetes mellitus, prolonged antibiotic use, poor nutritional status, and other conditions affecting host defenses, can promote *C. albicans* colonization and overgrowth. Local factors, including poor oral hygiene, xerostomia, altered oral microbiota, and dental prostheses, further facilitate fungal persistence and biofilm development. The interaction of these factors can compromise mucosal defenses and create a favorable environment for *C. albicans* colonization, ultimately contributing to the development and persistence of OC.

**Figure 4:**
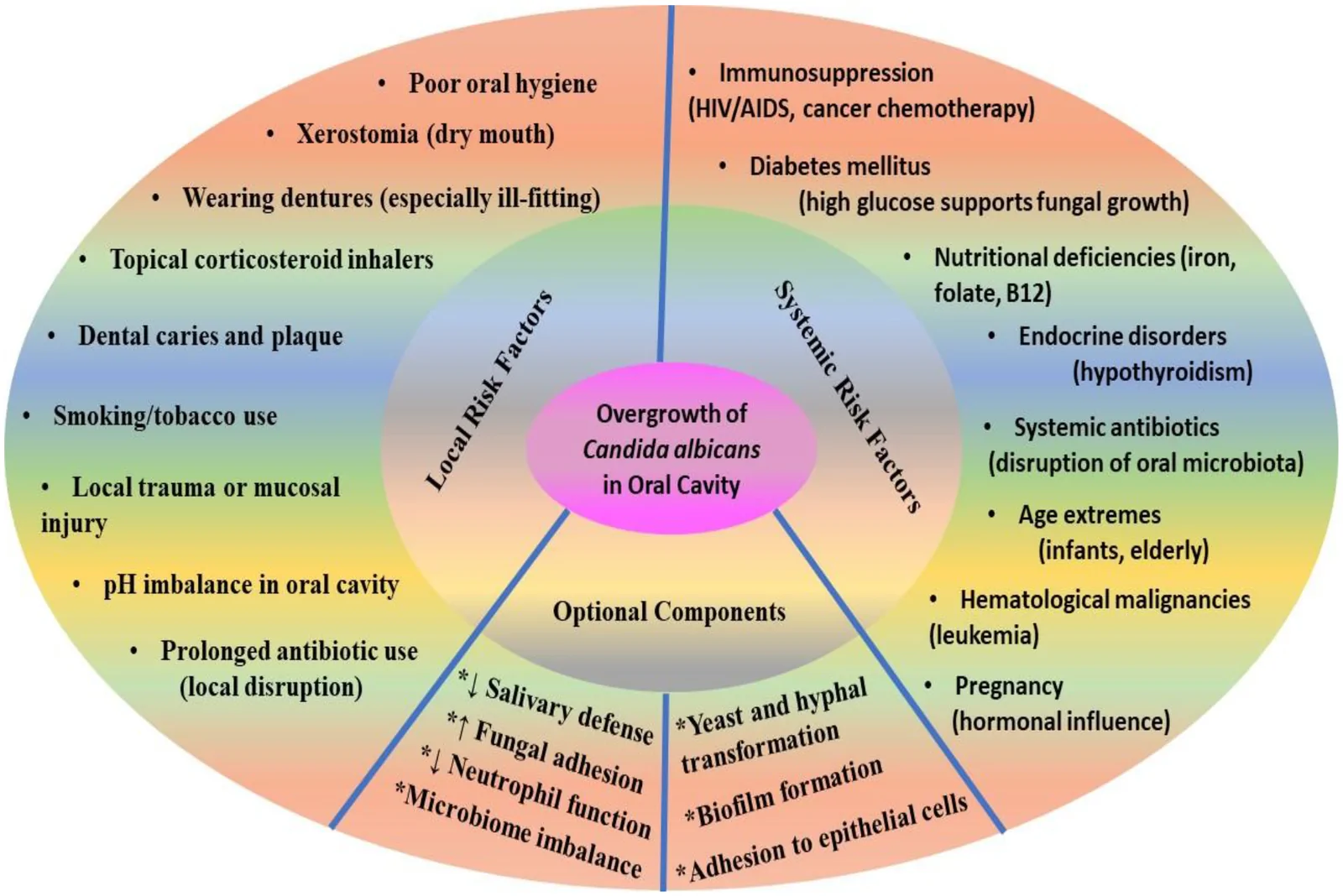
Factors affecting the pathogenesis of oral candidiasis.

The scientific literature identifies several local risk factors as contributors to OC. Poor oral hygiene allows microbial biofilms, including those containing *C. albicans*, to develop and reduces the ability to clear them from the oral mucosa. In particular, dental plaque, food debris, and other retained organic materials can provide nutrient-rich niches that support microbial growth and biofilm development, especially in the presence of additional predisposing factors (Di Cosola et al., 2021; Patel, 2022). Xerostomia, or dry mouth, is another important risk factor, as reduced saliva production can progressively deteriorate oral health. Specifically, decreased salivary secretion can reduce the availability of antimicrobial components, impair mechanical microbial clearance, and alter oral pH homeostasis, thereby facilitating *Candida* colonization (Patel, 2022). Dehydration, Sjogren’s syndrome, and many of the side effects from medications, including but not limited to antihistamines and antidepressants, play a major role in the reduction of saliva production, which increases infection risk (Akpan and Morgan, 2002; Patel, 2022; Sharma, 2019). Dental prostheses, such as poorly fitting dentures or those that aren’t cleaned effectively, may also contribute to the development of OC. As these devices develop biofilms, they may also cause localized trauma, increasing the likelihood of fungal colonization. The literature indicates that OC is multifactorial condition resulting from interactions among microbial, host, and environmental factors. However, it emphasizes the importance of reducing local risk factors by promoting good oral hygiene, managing dry mouth episodes, and maintaining proper denture care (Sardi et al., 2013; Sharma, 2019).

## Therapeutic advances in oral candidiasis

Antifungal agents used to treat OC include azoles, polyenes, echinocandins, and other formulations, all targeting different structures and functions of the fungal pathogen. Azoles, particularly miconazole, are frequently used for OC due to their broad-spectrum activity and ability to inhibit ergosterol synthase, thereby disrupting fungal cell membrane integrity (Das et al., 2024; Quindós et al., 2019; Santos et al., 2024). Matrix β-1,3 glucan is another important component of the EPS matrix that also sequesters antifungal drugs from the fungal cell wall, thus heightening observed resistance to fluconazole (Mallick et al., 2025; Mitchell et al., 2013). Miconazole, available as oral gels or lozenges, is particularly useful for localizing infection and, because of its sustained antifungal mechanism via direct contact with the oral mucosa, has very low systemic side effects (Sawant and Khan, 2017). In *C. albicans* biofilms, the transcription factor Efg1 provides an additional mode of resistance to miconazole, caspofungin, and amphotericin B (Bink et al., 2012).

Polyenes, nystatin, and amphotericin B act within the fungal cell membrane by binding ergosterol and forming pores that cause leakage of intracellular contents, ultimately leading to cell death. Because of decreased systemic absorption, nystatin is recommended for localized effects, whereas amphotericin B is reserved for severe cases due to its systemic toxicity (Carolus et al., 2020). Echinocandins (for example, caspofungin and micafungin) inhibit β-glucan in fungal cell walls and are most effective against azole-resistant species, including *C. glabrata* and *C. krusei*. Echinocandins are typically used for systemic infections but are also valuable in severe OC cases, especially in immunocompromised patients. Echinocandin resistance is minimal, although it is emerging in *C. glabrata* (Pfaller et al., 2019; Wiederhold, 2018). These antifungal agents demonstrate the need for personalized approaches to further address the growing problem of drug resistance (**Table 2**).

**Table 2:** Comparison of antifungal agents for oral candidiasis.

Class/Agent	Mechanism of action	Antifungal spectrum	Resistance profile	Clinical indications
Azoles	Inhibit lanosterol 14-α-demethylase, blocking ergosterol synthesis	Broad activity: *C. albicans*, some *C. glabrata*	Increasing resistance in *C. glabrata*, *C. krusei*, and biofilm-associated resistance	-First-line for mild-to-moderate OC -Prophylaxis in immunocompromised patients
Fluconazole	Fungistatic and interferes with ergosterol formation	*C. albicans*, *C. tropicalis*	High resistance in non-*albicans* species	-Mild to moderate OC -Effective in HIV-positive patients
Miconazole (topical)	-Same as fluconazole -Topical application	*C. albicans* infections	Less likely due to direct application	-Denture stomatitis, localized OC -Minimal systemic absorption
Polyenes	Bind to ergosterol in fungal membranes, forming pores	*C. albicans*, *C. tropicalis*, *C. parapsilosis*	Rare resistance; less effective against biofilms	Alternative to azoles; used topically or systemically depending on severity
Nystatin (topical)	-Fungicidal -Binds to ergosterol	Primarily *C. albicans*	Very low resistance due to minimal systemic use	Topical treatment for superficial OC in infants and denture wearers
Amphotericin *B*	-Fungicidal -Pore formation in the cell membrane	Broad-spectrum, including resistant *Candida* species	Emerging resistance in rare *Candida* species	Reserved for severe/refractory OC or systemic candidiasis
Echinocandins	Inhibit β-1,3-glucan synthase, disrupting cell wall synthesis	Broad *Candida* coverage, including azole-resistant strains	Low but increasing resistance (*C. glabrata*, *C. auris*)	Systemic OC in immunocompromised or azole-refractory cases
Caspofungin	Non-competitive inhibitor of β-1,3-glucan synthase	Strong against *C. albicans*, *C. glabrata*	Emerging FKS mutations	Parenteral use in resistant or disseminated OC

## Novel antifungals

Ibrexafungerp is a new triterpenoid antifungal and a promising treatment for *Candida* infections, particularly in patients with azole resistance. This antifungal inhibits 1, 3-β-D-glucan synthase, a critical enzyme involved in fungal cell wall synthesis. Studies indicate that Ibrexafungerp can treat multiple *Candida* isolates, including some that are azole-resistant, and it is considered safe with limited side effects. So far, it has been approved only for the treatment of vulvovaginal candidiasis, but studies are underway to investigate its use in other indications (Angulo et al., 2022; Jallow and Govender, 2021; Thakare et al., 2019).

Fosmanogepix is a novel antifungal that targets the *Candida* cell wall; however, it also inhibits the Gwt1 enzyme, which is involved in the biosynthesis of GPI-anchors. This mechanism results in cell death due to loss of cell wall structural integrity. In clinical trials, Fosmanogepix appears promising for treating infections caused by *Candida* species resistant to fluconazole, echinocandins, and polyenes. It is currently being studied in clinical trials, as it has the potential to offer a powerful new tool for treating invasive fungal infection caused by resistant fungal strains (Shaw and Ibrahim, 2020; Thakare et al., 2021).

Typical antifungal therapies aim to eliminate fungi, but a growing body of research focuses on boosting the host’s immune response to enhance the body’s ability to fight *C. albicans* infections. Host-directed therapies (HDT) aim to modulate the host’s immune response, enhancing innate and adaptive immunity and improving pathogen clearance. This area is especially necessary as antifungal resistance continues to become a greater concern (Contaldo et al., 2023; Lionakis et al., 2023).

## Immune modulation via Interleukin-17 pathway

One possible host-directed therapeutic strategy involves modulating immune pathways involved in the IL-17 axis, a key component of mucosal antifungal immunity a major proinflammatory cytokine. IL-17, a cytokine that is considered proinflammatory because it is produced by Th17 cells, which are critical in mucosal immunity, mainly in the defense against OC (Arzmi et al., 2014). Additionally, Th17 are key mediators of the immune response to *Candida* infections. The Th17 response recruits neutrophils to the site of infection, where mature neutrophils enhance antimicrobial peptide production and maintain epithelial barrier integrity, all of which work together to limit fungal growth. IL-17-secreting Th17 cells induce expression of fungal defense genes that regulate antifungal modules, producing defensins, cathelicidin, and antimicrobial peptides in the oral system (Gaffen et al., 2011; Hernández-Santos and Gaffen, 2012). Ongoing investigations are targeting and amplifying the IL-17 pathway to augment immune responses against OC. Research indicates that IL-17-deficient mice are more susceptible to *Candida* infections, underscoring IL-17’s role in clearing infections (Conti et al., 2009). Therefore, targeted modulation of the IL-17 pathway may have therapeutic potential for enhancing antifungal immunity in individuals with impaired host defenses, such as those with HIV or individuals with diabetes, who have an increased risk of developing chronic or recurrent OC (Conti and Gaffen, 2010; Gaffen et al., 2011; Hernández-Santos and Gaffen, 2012).

Among agents under investigation are IL-17 and IL-17 receptor agonists to elicit or enhance Th17 responses and improve fungal clearance. An agent that enhances or promotes IL-17 production or activity in the oral cavity may represent potential therapeutic options for patients with refractory or recurrent OC. Additionally, small molecules that will stimulate IL-17 production are being studied for their ability to restore immune responses against fungal pathogens (Conti and Gaffen, 2010; Gaffen et al., 2011; Hernández-Santos and Gaffen, 2012). In addition to modulating IL-17, other cytokine therapies that use interferons or tumor necrosis factor (TNF) are being examined for their ability to enhance immunity against *Candida* infections. However, a major challenge remains the precise regulation of cytokine activity to enhance antifungal defense without inducing excessive inflammation or immune-mediated tissue damage (Conti and Gaffen, 2015; Hernández-Santos and Gaffen, 2012). While modulation of IL-17 offers promising host-directed approaches, excessive or dysregulated IL-17 activation may also promote proinflammatory responses and contribute to chronic inflammation and tissue damage, particularly in individuals with pre-existing inflammatory or autoimmune conditions (Abusleme and Moutsopoulos, 2017; Gaffen and Moutsopoulos, 2020). Therefore, therapeutic strategies should aim for controlled and localized modulation of IL-17 rather than indiscriminate enhancement of this pathway. We also lack sufficient information regarding the long-term safety of augmenting pre-existing IL-17 pathways with therapeutic agents. Robust clinical trials are therefore required to establish the efficacy, appropriate therapeutic level, and long-term safety of IL-17-based interventions across diverse patient populations (Yamanaka-Takaichi et al., 2022).

## Photodynamic therapy in oral candidiasis

PDT is a non-invasive treatment that does not rely on systemic antifungal medications, thereby reducing the risks of side effects associated with oral or intravenous antifungal therapies (**Figure 5**), such as nephrotoxicity or hepatotoxicity (Contaldo et al., 2023). PDT is less likely to produce resistance than traditional antifungal agents. The ROS produced by PDT cause immediate and extensive cellular damage, making it unlikely that fungal cells can adapt or develop mechanisms to resist PDT (Baltazar et al., 2015; Li et al., 2021). PDT can act as an adjunct to conventional antifungal treatments, which can improve their effectiveness and reduce relapse (Dovigo et al., 2011). This could be very useful in cases of recurrent or chronic OC, when conventional antifungals may not be effective (Dovigo et al., 2011). Clinical studies have shown that PDT is well tolerated, with minimal adverse effects. PDT is considered safe and well-tolerated, with few reported side effects, including localized erythema (redness) or discomfort at the treatment site (Nunes et al., 2023).

**Figure 5:**
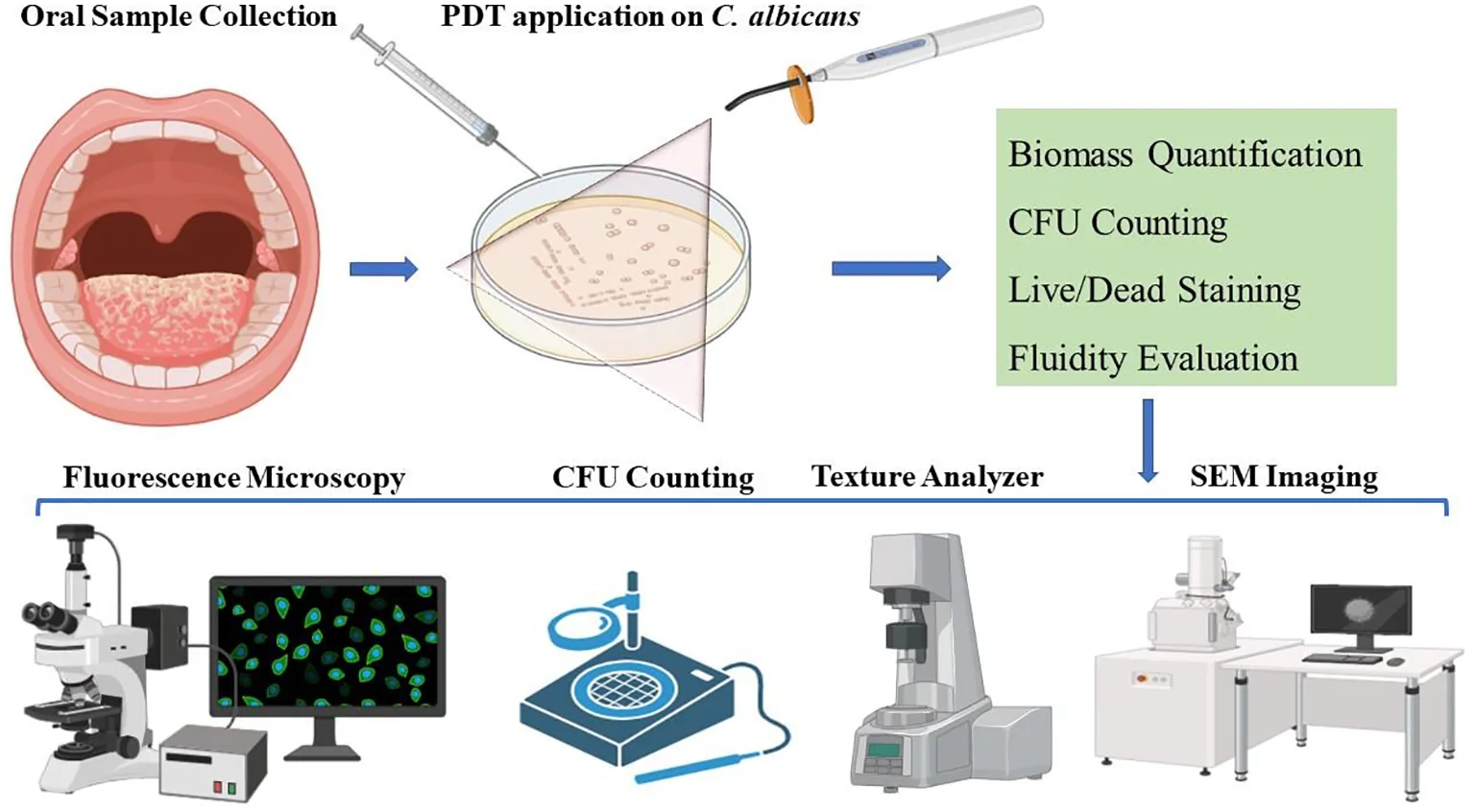
Experimental workflow for evaluating the effect of photodynamic therapy on *C. albicans* in oral candidiasis.

Several studies have investigated the efficacy of PDT for managing OC, particularly in immunocompromised patients and those with chronic and/or drug-resistant infections. In preclinical studies, PDT has shown a significant reduction in fungal burden, improved clinical outcomes, and faster healing of the oral mucosa (Alves et al., 2019). PDT has demonstrated certain benefits in managing patients with concomitant diseases, such as HIV/AIDS or diabetes, making patients predisposed to recurrent *Candida* infections, where conventional antifungal drugs fail (Javed et al., 2014). When comparing PDT solely with antifungal agents, improvements in therapeutic effect with fluconazole, for example, have primarily been attributed to increased permeability of the fungal cell wall, thereby limiting the dose and duration of treatment (Alves et al., 2017). PDT has also shown efficacy against fluconazole-resistant *C. albicans*, offering an additional management option that bypasses the typical mechanisms of antifungal drug resistance (Dovigo et al., 2011). The ROS generated during PDT can induce rapid and extensive cellular damage, thereby potentially reducing the likelihood of the development of resistance to PDT (Huang et al., 2018).

Clinical studies have also provided quantitative evidence supporting the potential of PDT. In a pilot clinical trial involving 21 adult AIDS patients with OC, two consecutive antimicrobial PDT treatments using methylene blue and potassium iodide produced a significant reduction in *C. albicans* viable counts after the second treatment and were accompanied by improvement in oral lesions and clinical symptoms (Du et al., 2021).In an earlier clinical study involving HIV-infected patients, PDT eliminated 100% of detectable *Candida* colonies, with no recurrence reported during the 30-day follow-up period (Scwingel et al., 2012). Furthermore, in a randomized clinical trial comparing PDT with topical nystatin for denture stomatitis, clinical success was observed in 45% of patients receiving PDT versus 53% of those receiving nystatin, while *Candida* colony counts were significantly reduced in both groups (Mima et al., 2012). In a randomized clinical trial of diabetic overdenture wearers, MB-PDT plus topical miconazole use resulted in lower *Candida* counts and improved patient-reported oral health quality of life compared with either treatment alone (de Senna et al., 2018). Similarly, Maciel et al. (2016) reported that 40% of patients treated with methylene blue-mediated PDT followed by low-level laser therapy achieved clinical and microbiological resolution, compared with an 80% cure rate in the miconazole group. At 15 days after treatment, recurrence occurred in 25% of the PDT-treated patients and 12.5% of miconazole-treated patients, demonstrating that treatment outcomes may vary depending on the PDT protocol used (Maciel et al., 2016). All of these findings indicate that PDT may provide a novel, adjunctive, and standalone treatment for challenging cases of OC. While PDT has antifungal potential, one of its main obstacles is the standardization of photosensitizers, dosing protocols, and light parameters. The variability across clinical studies limits reproducibility and contains the framework for creating guidelines for its use. In addition to variability, the cost of PDT, limited access to the therapy, and still-limited long-term follow-up data are significant barriers to routine practice.

## Probiotics in oral candidiasis treatment

Probiotics, especially *Lactobacillus* species, have been investigated as a potential adjunct to therapy for OC. In humans, *Lactobacillus* species are naturally occurring organisms that can help restore the oral microbiota and reduce the overgrowth of *C. albicans* (**Figure 6**) (Giordani et al., 2021). The mechanisms by which *Lactobacillus* species may inhibit *C. albicans* can be broadly categorized into the following:

**Figure 6:**
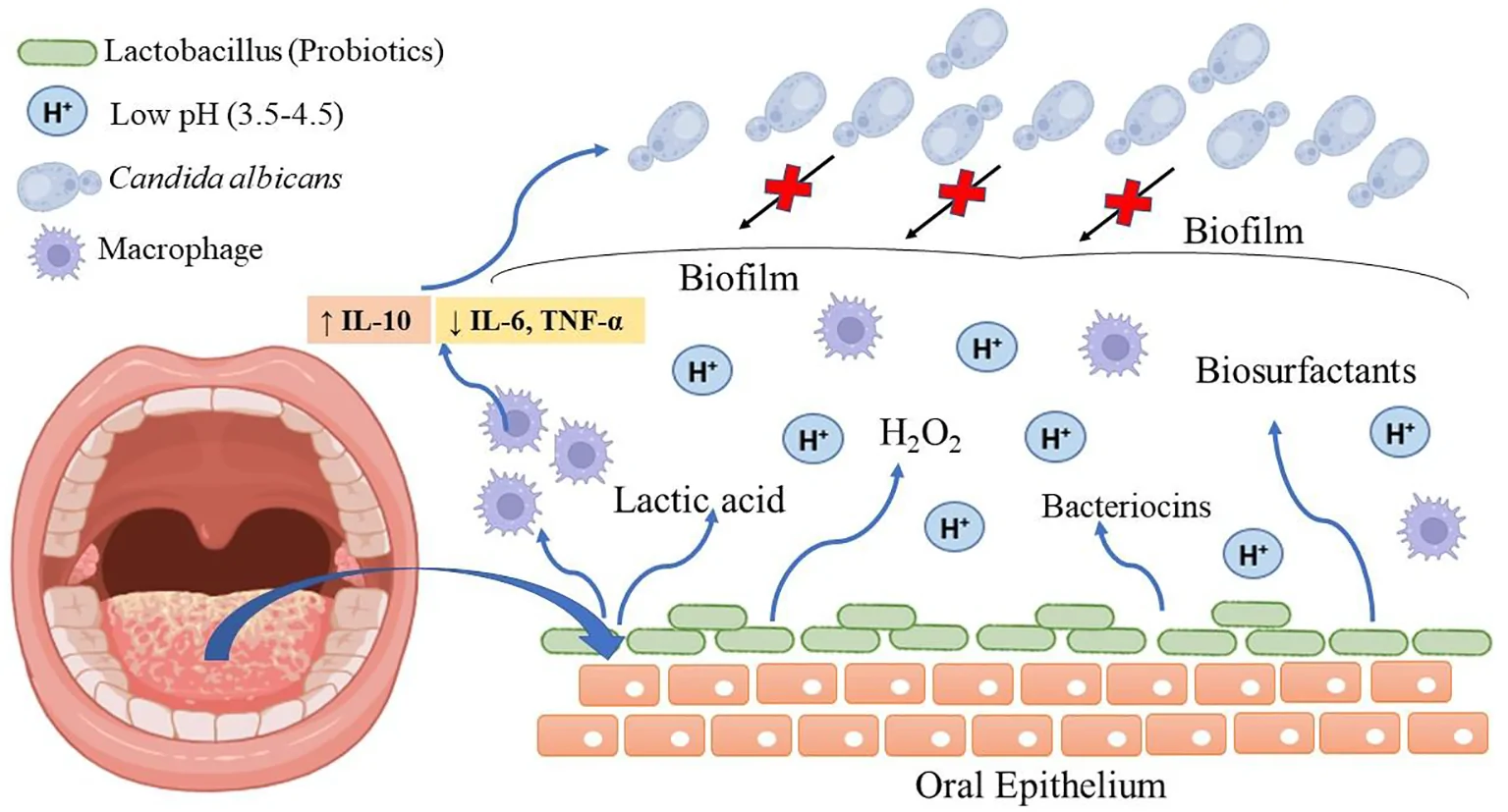
Mechanisms by which *Lactobacillus* probiotics inhibit *C. albicans* in the oral cavity. The protective mechanisms through which *Lactobacillus* probiotics can prevent OC mediated by *C. albicans*. Probiotics colonize the oral epithelium, and they exert antifungal actions by preventing the formation of fungal biofilm through mechanisms of competitive exclusion and by the production of antimicrobial substances (lactic acid, hydrogen peroxide (H_2_O_2_), bacteriocins, and surfactants) that acidify the immediate environment (pH 3.5-4.5), resulting in the inhibition of fungal growth. *Lactobacillus* can also modulate the host immune response by increasing the production of the anti-inflammatory cytokine IL-10 and decreasing the proinflammatory cytokines IL-6 and TNF-α; thereby suppressing the proinflammatory response and assisting in the maintenance of mucosal immunity. This complement of protective factors supports the maintenance of the oral microbial balance and prevents the overgrowth of *C. albicans*.

### Competitive exclusion and inhibition of adhesion

One of the ways that *Lactobacillus* species inhibit colonization of *Candida* is through competition exclusion (Zeise et al., 2021). *L. rhamnosus* and *L. reuteri* can adhere to epithelial surfaces of the oral cavity, blocking the sites available for *Candida* for adhesion. This process reduces the opportunity for *Candida* to colonize and form biofilms, thereby reducing the risk of infection and antifungal resistance (Mundula et al., 2019).

### Acidification of the oral environment

*Lactobacilli* also lower the pH of the oral cavity by producing lactic acid. The low pH also inhibits the growth of several invasive pathogens, including *C. albicans*, which promotes growth at neutral pH.

### Production of antimicrobial compounds

In addition, *Lactobacillus* species produce bacteriocins and hydrogen peroxide, both of which are antimicrobial and inhibit *Candida* growth (Matsubara et al., 2016). These antimicrobial metabolites, together with lactic acid and other secreted compounds, may contribute to suppression of *Candida* growth and colonization.

### Inhibition and disruption of biofilm formation

One significant obstacle in treating OC is *Candida* biofilms’ ability to protect cells from the host immune response and antifungal agents. *Lactobacillus* species can limit biofilm formation and may also disrupt pre-existing biofilms by producing metabolites that degrade the ECM or inhibit its formation. *L. casei* and *L. rhamnosus* have been shown to decrease biofilm formation and promote the detachment of *Candida* cells from surfaces, which enhances the efficacy of antifungal therapies (Song and Lee, 2017). Thus, the antibiofilm activity of probiotics may represent an important complementary mechanism for controlling persistent *Candida* infections.

### Modulation of the host immune response

*Lactobacillus* species also modulate the host immune response, including by promoting the release of cytokines that activate dendritic cells, macrophages, and T cells; these immune responses can further enhance host recognition and specificity in eliminating *Candida* cells. *Lactobacillus* can also enhance the actions of neutrophils and other phagocytic cells, which are essential for *Candida* clearance (Zangl et al., 2019; Zeise et al., 2021). As illustrated in **Figure 6**, *Lactobacillus* may also increase the production of the anti-inflammatory cytokine IL-10 while decreasing proinflammatory cytokines such as IL-6 and TNF-α, thereby supporting mucosal immune homeostasis.

### Clinical evidence for probiotic efficacy

Both studies by Ishikawa et al. (2015) and Miyazima et al. (2017) reported that *Lactobacillus*-based probiotics effectively reduced oral *Candida* colonization in elderly denture wearers. In a prospective observational study, Elsayes et al. (2024) confirmed the prophylactic effects of a single brand of multispecies probiotic lozenges in geriatric cohorts who were wearing their dentures for the first time. The 2019 meta-analysis by Hu et al. confirmed the clinical efficacy and safety of probiotic use, while highlighting the need for higher-quality trials (Hu et al., 2019). Rodrigues-Arias et al. (2022) concluded that specific selected strains of *Lactobacillus* could inhibit *C. albicans* during the early stages of adhesion in yeast, therefore demonstrating some level of competition exclusion. Another clinical study by Rajão et al. (2025), observed *that Limosilactobacillus reuteri* AJCR4 effectively disrupted *Candida* biofilms, particularly on surfaces that mimic oral conditions, suggesting that *L. reuteri* may be used for denture-related candidiasis in the oral cavity. Jiang et al. (2015) investigated the inhibitory effects of probiotic lactobacilli metabolic by-products, which produced by probiotic lactobacilli, including those generated under fermentation conditions. In a clinical context, Kraft-Bodi et al. (2015) showed that elderly individuals receiving *L. reuteri* probiotic lozenges had a statistically significant decrease in oral *Candida* colonization compared with the placebo group. Allonsius et al. (2017) provided further mechanistic evidence indicating that *L. rhamnosus* GG exerted direct anti-*Candida* effects through exopolysaccharides, which inhibited hyphal formation and adhesive behavior. These findings support the potential of strain-specific *Lactobacillus*-based probiotics as adjunctive or preventive therapies against OC, particularly among high-risk groups such as denture wearers and immunocompromised individuals. However, differences in probiotic strains, formulations, dosage, and treatment duration highlight the need for standardized clinical studies.

## Complementary and integrative therapeutic approaches in oral candidiasis

The increasing prevalence of antifungal resistance and the remarkable tolerance of *C. albicans* biofilms have stimulated interest in complementary and integrative therapeutic strategies that can be used alongside conventional antifungal agents (Debta et al., 2026; Mallick et al., 2026; Sahu et al., 2026a, 2026b). Natural products, phytotherapeutics, essential oils, phenolic compounds, nanoparticles, antimicrobial peptides, and rationally combined therapies have demonstrated promising antifungal and antibiofilm properties. They may offer additional approaches for managing persistent or recurrent OC. Importantly, these approaches should currently be considered as potential adjuncts rather than replacements for established antifungal therapy, because much of the available evidence remains preclinical or comes from small-scale clinical studies.

Natural products and phytotherapeutics represent an important source of structurally diverse antifungal compounds. Medicinal plant extracts and their bioactive constituents have demonstrated inhibitory effects against *Candida* growth, adhesion, hyphal development, and biofilm formation. Several compounds, including curcumin, cinnamaldehyde, eugenol, carvacrol, thymol, terpinen-4-ol, linalool, geraniol, epigallocatechin gallate, limonene, citral, and related phytochemicals, have been investigated for their ability to interfere with different stages of *Candida* biofilm development (Sahu et al., 2026c; Shariati et al., 2022). These compounds may act through multiple mechanisms, including disruption of fungal membrane integrity, interference with ergosterol-associated processes, inhibition of morphogenesis, attenuation of adhesion, and modulation of extracellular biofilm matrix formation. Such multitarget effects are particularly attractive because they may reduce the ability of *C. albicans* to compensate via a single resistance mechanism (Shankar Raut and Mohan Karuppayil, 2016; Shariati et al., 2022).

Essential oils and their major constituents represent another promising phytotherapeutic strategy for OC. Oils derived from medicinal plants, including garlic, cinnamon, lemongrass, tea tree, thyme, clove, and other aromatic plants, have demonstrated activity against *Candida* oral biofilms (Almeida et al., 2016; Bersan et al., 2014; Guimarães et al., 2021). Their lipophilic constituents can interact with fungal membranes and compromise cellular integrity, while some also affect adhesion, hyphal development, and biofilm maturation (Nazzaro et al., 2017; Zahraoui, 2025). A review of medicinal plants used against oral *Candida* biofilms identified several essential oils with substantial antibiofilm activity, particularly those derived from *Allium sativum*, *Cinnamomum zeylanicum*, and *Cymbopogon citratus* (Guimarães et al., 2021). Recent evidence further indicates that combinations of essential oils may produce stronger antibiofilm effects than individual oils, supporting the concept that multi-component natural products can exert complementary or synergistic effects. For example, tea tree and Ceylon cinnamon bark oils demonstrated strong synergistic activity against *C. albicans* biofilms, with a fractional inhibitory concentration index (FICI) of 0.375. At the same time, optimized essential-oil combinations achieved approximately 89-94% inhibition of 24-h *Candida* biofilms (Balázs et al., 2026).

Phenolic compounds are particularly relevant because many essential oils and medicinal plants contain phenolic or phenolic-like constituents with potent antifungal and antibiofilm activities. Compounds such as eugenol, thymol, carvacrol, cinnamaldehyde, and curcumin have been reported to interfere with *Candida* biofilm formation and virulence (Braga et al., 2008; Didehdar et al., 2022; Molaeitabari and Dahms, 2025; Priya and Pandian, 2022; Shariati et al., 2022; Tuan and Masak, 2026). Their activity may involve disruption of fungal membrane integrity, oxidative stress, interference with morphogenesis, and inhibition of biofilm-associated processes. The broad range of natural antibiofilm compounds reported against *Candida* suggests that targeting virulence and biofilm development, rather than fungal viability alone, may represent a useful complementary strategy (Shariati et al., 2022).

Nanotechnology-based approaches have also attracted considerable interest because nanoparticles can interact directly with fungal cells and biofilm matrices and can be combined with conventional antifungal agents. Zinc oxide nanoparticles (ZnO NPs), for example, have demonstrated inhibitory activity against *C. albicans* biofilms and virulence-associated processes (Sharma and Ghose, 2015). Biogenic ZnO NPs have been shown to inhibit *C. albicans* growth, morphogenesis, and biofilm formation, with molecular studies demonstrating modulation of genes involved in virulence and morphogenesis, including *efg*1, *hwp*1, *ras*1, *als*3, *bcy*1, *nrg*1, and *tup*1 (Joshi et al., 2022). Other ZnO-based nanomaterials have also demonstrated antifungal activity and biofilm inhibition, while green-synthesized ZnO NPs have shown synergistic effects when combined with conventional agents such as fluconazole, nystatin, or terbinafine (Mathew et al., 2025; Yassin et al., 2025, 2023). These findings suggest that nanoparticles may function not only as direct antifungal agents but also as potentiators of conventional therapy. However, nanoparticle size, surface chemistry, dose, synthesis method, tissue compatibility, and long-term toxicity must be carefully evaluated before clinical translation.

Antimicrobial peptides offer a complementary strategy, as they can target fungal cells and biofilms through mechanisms distinct from those of conventional azoles and polyenes (Morici et al., 2016; Sun et al., 2022). Oral and host-derived peptides may interfere with fungal membrane function, adhesion, biofilm development, or cell-cell interactions. Statherin-derived peptides, for example, have been investigated for their effects on *C. albicans* metabolism and biofilm development, highlighting the potential of salivary-associated peptides as locally relevant antifungal approaches (Pellissari et al., 2021). More recently, anti-biofilm peptides such as Pom-1 and its derivatives have demonstrated activity against *C. albicans* biofilms and have restored the efficacy of fluconazole and amphotericin B against resistant isolates when used in combination (Kissmann et al., 2025). This finding is particularly important because it demonstrates how a biofilm-targeting agent can complement rather than duplicate the mechanism of conventional antifungal drugs (Kissmann et al., 2025).

Combination therapy represents a broader integrative strategy in which agents with complementary mechanisms are used to improve antifungal efficacy, reduce required drug concentrations, and potentially limit the emergence of resistance. Natural products and essential oils have demonstrated synergistic or additive interactions with azoles and other antifungal agents (Parker et al., 2022; Tullio et al., 2019). For instance, essential oils have enhanced the activity of fluconazole and other antifungal drugs against *Candida* biofilms. At the same time, plant-derived compounds may increase fungal membrane permeability, interfere with efflux mechanisms, or affect virulence pathways, thereby increasing susceptibility to conventional antifungal agents. Recent evidence also supports the potential of combining phytochemicals with conventional antifungals via multitarget mechanisms including membrane disruption, efflux-pump inhibition, mitochondrial dysfunction, and oxidative stress (Sharma et al., 2026; Tuan and Masak, 2026). Therefore, combinations involving phytotherapeutics, essential oils, nanoparticles, AMPs, probiotics, or PDT may provide complementary effects against different stages of *C. albicans* infection, particularly biofilm-associated disease.

Despite these encouraging findings, most natural-product, nanoparticle, and peptide-based interventions remain at the experimental stage. Differences in extraction procedures, chemical composition, nanoparticle physicochemical characteristics, peptide sequence, concentration, formulation, and experimental models make comparisons between studies difficult. In addition, potential cytotoxicity, mucosal irritation, pharmacokinetic properties, interactions with the oral microbiome, and long-term safety require careful investigation. Future clinical studies should therefore establish standardized formulations, clinically relevant dosing regimens, safety profiles, and well-defined synergistic interactions with conventional antifungals. A rational combination of conventional antifungal therapy with complementary antibiofilm approaches may provide a more effective strategy for managing recurrent, resistant, and biofilm-associated OC.

## Antibiofilm strategies and clinical perspectives

Biofilm formation represents one of the major therapeutic challenges in OC, because *C. albicans* biofilms exhibit substantially greater tolerance to antifungal agents than planktonic cells. The three-dimensional biofilm architecture, phenotypic heterogeneity, efflux activity, persister cells, and ECM collectively protect fungal cells from antifungal drugs and host immune responses. In particular, the ECM contains polysaccharides such as β-1, 3-glucan, α-mannan, and β-1, 6-glucan, which contribute to biofilm structural integrity and can sequester antifungal agents, thereby reducing their effective concentration within the biofilm (Cavalheiro and Teixeira, 2018; Nett and Andes, 2020). This biofilm-associated tolerance is particularly relevant to recurrent OC, where residual biofilm-associated fungal cells may survive antifungal treatment and subsequently repopulate the oral mucosa, contributing to persistent or recurrent infection.

Consequently, therapeutic strategies that specifically target the biofilm rather than fungal viability alone are gaining increasing attention. One promising approach is ECM disruption, particularly targeting β-1, 3-glucan. β-1, 3-glucanase has been shown to disrupt *C. albicans* biofilms and increase susceptibility to fluconazole and amphotericin B, demonstrating the potential of matrix-degrading enzymes as adjunctive agents. Other emerging strategies include quorum-sensing inhibition, interference with adhesion and hyphal development, efflux-pump modulation, natural compounds, antimicrobial peptides, nanoparticles, and drug-delivery systems designed to improve antifungal penetration into mature biofilms (Atriwal et al., 2021). Importantly, antibiofilm therapy may be most effective when combined with conventional antifungal treatment. Rather than relying exclusively on higher antifungal doses, matrix-disrupting or biofilm-weakening agents can increase drug penetration and restore antifungal susceptibility. Recent studies further support the development of ECM-directed adjunctive therapies, while natural compounds and nanotechnology-enabled drug-delivery systems are being explored to improve antifungal exposure within biofilms (Tuan and Masak, 2026). Therefore, future clinical management of recurrent or refractory OC may benefit from biofilm-directed combination therapy, in which conventional antifungals are combined with agents targeting the ECM, adhesion, morphogenesis, quorum sensing, or other biofilm-specific mechanisms. However, most antibiofilm approaches remain at the experimental or early translational stage, and further clinical studies are required to establish their safety, optimal dosing, efficacy against mature oral biofilms, and effectiveness in preventing recurrence.

## Future directions for addressing challenges in oral candidiasis management

Effectively controlling OC will require novel interventional approaches to overcome the limitations of existing treatments, including antimicrobial resistance and limited access to safe treatments. Exploring new classes of antifungal agents with unique mechanisms of action, remains a central focus, as these drugs may help tackle resistant *Candida* strains and improve patient outcomes (Angulo et al., 2022; Thakare et al., 2021). In addition to directly targeting fungal viability, future therapeutic strategies may increasingly focus on anti-virulence approaches that interfere with key pathogenic traits, including adhesion, hyphal transition, biofilm formation, quorum sensing, and host–pathogen interactions. By targeting virulence rather than fungal viability alone, such approaches may exert reduced selective pressure for the development of antifungal resistance. Other approaches, such as host-directed therapies that enhance host immune defenses, will be interesting as alternatives or adjuncts to conventional antifungal therapy. However, the efficacy and safety of this approach will require further preclinical and clinical study.

Emerging gene-editing technologies, including CRISPR/Cas-based approaches, may provide powerful tools for identifying and validating genes involved in *C. albicans* virulence, antifungal resistance, and biofilm formation. Such technologies could improve mechanistic understanding and facilitate the identification of novel therapeutic targets. Similarly, artificial intelligence (AI) and machine-learning approaches may accelerate antifungal drug discovery by predicting drug–target interactions, identifying potential antifungal compounds, repurposing existing drugs, and prioritizing promising candidates for experimental validation.

Newer approaches, such as PDT, will require rigorous standardization of photosensitizer selection, light-delivery parameters, and treatment duration before they can be translated into routine clinical practice; otherwise, variability in treatment protocols may compromise therapeutic efficacy and clinical reproducibility. Meanwhile, probiotics that employ *Lactobacillus* species require further research to optimize strain selection, dosage, formulation, and delivery methods. The oral microbiome should also be considered in future therapeutic development because disruption of the normal microbial community can influence *Candida* colonization and disease progression. Strategies that preserve or restore a healthy oral microbial ecosystem, including carefully selected probiotics and microbiome-directed interventions, complement antifungal therapy. Personalized medicine may provide a more effective framework for managing recurrent or refractory OC by integrating fungal species identification and resistance profiles with host characteristics, immune status, oral microbiome composition, and clinical risk factors. Combining these approaches with traditional treatments and clinical biomarker phenotyping may help overcome the overall challenges in OC, particularly in high-risk and immunocompromised patients.

## Conclusion

*C. albicans* continues to be a major etiological agent in OC, particularly among immunocompromised populations. Its ability to form biofilms, undergo morphological transitions, evade host immunity, and resist antifungal agents underscores the complexity of its pathogenesis. Traditional antifungal treatments, while effective in many cases, face limitations due to increasing resistance and biofilm-associated tolerance. The reviewed evidence indicates that the cAMP-PKA, MAPK, Rim101, and TOR signaling pathways play important roles in regulating fungal morphogenesis, virulence, and biofilm development, making these pathways potential targets for future therapeutic interventions. Recent advances in immune-modulating therapies targeting IL-17, as well as probiotic and photodynamic approaches, offer promising complementary strategies. Furthermore, natural products, phytotherapeutics, essential oils, phenolic compounds, nanoparticles, and antimicrobial peptides show potential as antibiofilm or adjunctive approaches, particularly when combined with conventional antifungal agents. Because biofilm-associated persistence is an important contributor to treatment failure and recurrence, future therapeutic development should increasingly focus on disrupting the extracellular matrix and other biofilm-associated mechanisms. Future research should focus on rational combination therapies, anti-virulence and antibiofilm strategies, resistance monitoring, oral microbiome-directed interventions, and personalized treatment based on host-pathogen interactions to optimize clinical outcomes.

## References

[B1] Abusleme L. Moutsopoulos N. M. (2017). IL‐17: overview and role in oral immunity and microbiome. Oral. Dis. 23, 854–865. doi: 10.1111/odi.12598 27763707 PMC5398954

[B2] Ahmad P. Akhtar U. Chaudhry A. Rahid U. Saif S. Asif J. A. (2019). Repercussions of diabetes mellitus on the oral cavity. Eur. J. Gen. Dentistry 8, 55–62. doi: 10.4103/ejgd.ejgd_28_1942621334

[B3] Akpan A. Morgan R. (2002). Oral candidiasis. Postgrad. Med. J. 78, 455–459. doi: 10.1136/pmj.78.922.455 12185216 PMC1742467

[B4] Allonsius C. N. van den Broek M. F. De Boeck I. Kiekens S. Oerlemans E. F. Kiekens F. . (2017). Interplay between *Lactobacillus rhamnosus* GG and *Candida* and the involvement of exopolysaccharides. Microb. Biotechnol. 10, 1753–1763. doi: 10.1111/1751-7915.12799 28772020 PMC5658588

[B5] Almeida L. D. F. D. D. Paula J. F. D. Almeida R. V. D. D. Williams D. W. Hebling J. Cavalcanti Y. W. (2016). Efficacy of citronella and cinnamon essential oils on *Candida albicans* biofilms. Acta Odontol. Scand. 74, 393–398. doi: 10.3109/00016357.2016.1166261 27098375

[B6] Alves F. Carmello J. C. Mima E. G. D. O. Costa C. A. D. S. Bagnato V. S. Pavarina A. C. (2019). Photodithazine-mediated antimicrobial photodynamic therapy against fluconazole-resistant *Candida albicans in vivo*. Med. Mycol. 57, 609–617. doi: 10.1093/mmy/myy083 30339223

[B7] Alves F. de Oliveira Mima E. G. Passador R. C. P. Bagnato V. S. Jorge J. H. Pavarina A. C. (2017). Virulence factors of fluconazole-susceptible and fluconazole-resistant *Candida albicans* after antimicrobial photodynamic therapy. Lasers Med. Sci. 32, 815–826. doi: 10.1007/s10103-017-2177-y 28280998

[B8] Angulo D. A. Alexander B. Rautemaa-Richardson R. Alastruey-Izquierdo A. Hoenigl M. Ibrahim A. S. . (2022). Ibrexafungerp, a novel triterpenoid antifungal in development for the treatment of mold infections. J. Fungi 8, 1121. doi: 10.3390/jof8111121 36354888 PMC9695964

[B9] Arzmi M. Alshwaimi E. Harun W. W. Razak F. A. Farina F. McCullough M. . (2014). Gaining more insight into the determinants of *Candida* species pathogenicity in the oral cavity. SAGE Publications SAGE UK: London Engl. 12, 227–235.

[B10] Atriwal T. Azeem K. Husain F. M. Hussain A. Khan M. N. Alajmi M. F. . (2021). Mechanistic understanding of *Candida albicans* biofilm formation and approaches for its inhibition. Front. Microbiol. 12, 638609. doi: 10.3389/fmicb.2021.638609 33995297 PMC8121174

[B11] Balázs V. L. Puskár L. Ormai E. Farkas Á. Szilágyi-Utczás M. Kocsis B. . (2026). Synergistic anti-*Candida* effects of essential oil blends on biofilms at various maturation stages. Eur. J. Pharm. Sci. 221, 107515. doi: 10.1016/j.ejps.2026.107515 41871816

[B12] Baltazar L. M. Ray A. Santos D. A. Cisalpino P. S. Friedman A. J. Nosanchuk J. D. (2015). Antimicrobial photodynamic therapy: an effective alternative approach to control fungal infections. Front. Microbiol. 6, 202. doi: 10.3389/fmicb.2015.00202 25821448 PMC4358220

[B13] Bersan S. M. Galvão L. C. Goes V. F. Sartoratto A. Figueira G. M. Rehder V. L. . (2014). Action of essential oils from Brazilian native and exotic medicinal species on oral biofilms. BMC Complementary Altern. Med. 14, 451. doi: 10.1186/1472-6882-14-451 25407737 PMC4289052

[B14] Bink A. Govaert G. Vandenbosch D. Kuchariková S. Coenye T. Nelis H. . (2012). Transcription factor Efg1 contributes to the tolerance of *Candida albicans* biofilms against antifungal agents *in vitro* and *in vivo*. J. Med. Microbiol. 61, 813–819. doi: 10.1099/jmm.0.041020-0 22422573

[B15] Biswas S. Van Dijck P. Datta A. (2007). Environmental sensing and signal transduction pathways regulating morphopathogenic determinants of *Candida albicans*. Microbiol. Mol. Biol. Rev. 71, 348–376. doi: 10.1128/mmbr.00009-06 17554048 PMC1899878

[B16] Bojang E. Ghuman H. Kumwenda P. Hall R. A. (2021). Immune sensing of *Candida albicans*. J. Fungi 7, 119. doi: 10.3390/jof7020119 33562068 PMC7914548

[B17] Bondaryk M. Kurzątkowski W. Staniszewska M. (2013). Antifungal agents commonly used in the superficial and mucosal candidiasis treatment: mode of action and resistance development. Adv. Dermatol. Allergology/Postępy Dermatologii i Alergologii 30, 293–301. doi: 10.5114/pdia.2013.38358 24353489 PMC3858657

[B18] Braga P. C. Culici M. Alfieri M. Dal Sasso M. (2008). Thymol inhibits Candida albicans biofilm formation and mature biofilm. Int. J. Antimicrob. Agents 31, 472–477. doi: 10.1016/j.ijantimicag.2007.12.013 18329858

[B19] Braunsdorf C. Mailänder‐Sánchez D. Schaller M. (2016). Fungal sensing of host environment. Cell. Microbiol. 18, 1188–1200. doi: 10.1111/cmi.12610 27155351

[B20] Carolus H. Pierson S. Lagrou K. Van Dijck P. (2020). Amphotericin B and other polyenes—discovery, clinical use, mode of action and drug resistance. J. Fungi 6, 321. doi: 10.3390/jof6040321 33261213 PMC7724567

[B21] Cavalheiro M. Teixeira M. C. (2018). *Candida* biofilms: threats, challenges, and promising strategies. Front. Med. 5, 28. doi: 10.3389/fmed.2018.00028 29487851 PMC5816785

[B22] Černáková L. Rodrigues C. F. (2020). Microbial interactions and immunity response in oral *Candida* species. Future Microbiol. 15, 1653–1677. doi: 10.2217/fmb-2020-0113 33251818

[B23] Chow E. W. L. Pang L. M. Wang Y. (2021). From Jekyll to Hyde: the yeast–hyphal transition of *Candida albicans*. Pathogens 10, 859. doi: 10.3390/pathogens10070859 34358008 PMC8308684

[B24] Contaldo M. Di Stasio D. Romano A. Fiori F. Della Vella F. Rupe C. . (2023). Oral candidiasis and novel therapeutic strategies: Antifungals, phytotherapy, probiotics, and photodynamic therapy. Curr. Drug Delivery 20, 441–456. doi: 10.2174/1567201819666220418104042 35440307

[B25] Conti H. R. Gaffen S. L. (2010). Host responses to *Candida albicans*: Th17 cells and mucosal candidiasis. Microbes Infection 12, 518–527. doi: 10.1016/j.micinf.2010.03.013 20381638 PMC2892252

[B26] Conti H. R. Gaffen S. L. (2015). IL-17–Mediated immunity to the opportunistic fungal pathogen *Candida albicans*. J. Immunol. 195, 780–788. doi: 10.4049/jimmunol.1500909 26188072 PMC4507294

[B27] Conti H. R. Shen F. Nayyar N. Stocum E. Sun J. N. Lindemann M. J. . (2009). Th17 cells and IL-17 receptor signaling are essential for mucosal host defense against oral candidiasis. J. Exp. Med. 206, 299–311. doi: 10.1084/jem.20081463 19204111 PMC2646568

[B28] Cordeiro R. D. A. Teixeira C. E. Brilhante R. S. Castelo-Branco D. S. Alencar L. P. de Oliveira J. S. . (2015). Exogenous tyrosol inhibits planktonic cells and biofilms of *Candida* species and enhances their susceptibility to antifungals. FEMS Yeast Res. 15, fov012. doi: 10.1093/femsyr/fov012 25795651

[B29] Czajka K. M. Venkataraman K. Brabant-Kirwan D. Santi S. A. Verschoor C. Appanna V. D. . (2023). Molecular mechanisms associated with antifungal resistance in pathogenic Candida species. Cells 12 (22), 2655. doi: 10.3390/cells12222655 PMC10670235 37998390

[B30] Das S. Mallick U. Sahu B. K. Turuk J. Sahu M. C. (2024). Clinical mycology: Understanding pathogenesis, diagnosis, and antifungal strategies for invasive fungal infections: A review. Microbes Infect. Dis. 7, 713–734. doi: 10.21608/MID.2024.331599.2319

[B31] Debta P. Sahu B. K. Patra S. K. Debta F. M. Mishra E. Panda S. K. (2026). Overcoming *Candida* biofilm resistance: targeting persister cells with probiotic-derived metabolites. Front. Antibiot. 5, 1767028. doi: 10.3389/frabi.2026.1767028 41815221 PMC12972748

[B32] Debta P. Swain S. K. Sahu M. C. Debta F. M. Mohanty J. N. (2020). A review on oral *Candida* as commensal and opportunistic pathogen. Indian J. Forensic Med. Toxicol. 14, 8381–8388.

[B33] de Groot P. W. Bader O. de Boer A. D. Weig M. Chauhan N. (2013). Adhesins in human fungal pathogens: glue with plenty of stick. Eukaryotic Cell. 12, 470–481. doi: 10.1128/ec.00364-12 23397570 PMC3623432

[B34] de Senna A. M. Vieira M. M. MaChado-de-Sena R. M. Bertolin A. O. Núñez S. C. Ribeiro M. S. (2018). Photodynamic inactivation of *Candida* ssp. on denture stomatitis. A clinical trial involving palatal mucosa and prosthesis disinfection. Photodiagn. Photodyn. Ther. 22, 212–216. doi: 10.1016/j.pdpdt.2018.04.008 29678677

[B35] Dhillon N. K. Sharma S. Khuller G. (2003). Signaling through protein kinases and transcriptional regulators in *Candida albicans*. Crit. Rev. Microbiol. 29, 259–275. doi: 10.1080/713610451 14582620

[B36] Di Cosola M. Cazzolla A. P. Charitos I. A. Ballini A. Inchingolo F. Santacroce L. (2021). *Candida albicans* and oral carcinogenesis. A brief review. J. Fungi 7, 476. doi: 10.3390/jof7060476 34204731 PMC8231483

[B37] Didehdar M. Chegini Z. Shariati A. (2022). Eugenol: A novel therapeutic agent for the inhibition of *Candida* species infection. Front. Pharmacol. 13, 872127. doi: 10.3389/fphar.2022.872127 36016558 PMC9395595

[B38] Dovigo L. N. Pavarina A. C. de Oliveira Mima E. G. Giampaolo E. T. Vergani C. E. Bagnato V. S. (2011). Fungicidal effect of photodynamic therapy against fluconazole‐resistant *Candida albicans* and *Candida glabrata*. Mycoses 54, 123–130. doi: 10.1111/j.1439-0507.2009.01769.x 19889173

[B39] Du M. Xuan W. Zhen X. He L. Lan L. Yang S. . (2021). Antimicrobial photodynamic therapy for oral *Candida* infection in adult AIDS patients: A pilot clinical trial. Photodiagn. Photodyn. Ther. 34, 102310. doi: 10.1016/j.pdpdt.2021.102310 33901690

[B40] Egusa H. Soysa N. S. Ellepola A. N. Yatani H. Samaranayake L. P. (2008). Oral candidosis in HIV-infected patients. Curr. HIV Res. 6, 485–499. doi: 10.2174/157016208786501445 18991614

[B41] Elsayes S. A. El Attar M. S. ElHadary A. Aboulela A. G. Essawy M. M. Soliman I. S. (2024). Antimycotic prophylaxis with multispecies probiotics against oral candidiasis in new complete denture wearers: A randomized clinical trial. J. Prosthetic Dentistry 134, 1852–1859. doi: 10.1016/j.prosdent.2024.05.018 38906771

[B42] Fanning S. Mitchell A. P. (2012). Fungal biofilms. PloS Pathog. 8, e1002585. doi: 10.1371/journal.ppat.1002585 22496639 PMC3320593

[B43] Gaffen S. L. Hernández-Santos N. Peterson A. C. (2011). IL-17 signaling in host defense against *Candida albicans*. Immunol. Res. 50, 181–187. doi: 10.1007/s12026-011-8226-x 21717069 PMC3257840

[B44] Gaffen S. L. Moutsopoulos N. M. (2020). Regulation of host-microbe interactions at oral mucosal barriers by type 17 immunity. Sci. Immunol. 5, eaau4594. doi: 10.1126/sciimmunol.aau4594 31901072 PMC7068849

[B45] Giordani B. Parolin C. Vitali B. (2021). Lactobacilli as anti-biofilm strategy in oral infectious diseases: a mini-review. Front. Med. Technol. 3, 769172. doi: 10.3389/fmedt.2021.769172 35047965 PMC8757881

[B46] Gow N. A. Netea M. G. Munro C. A. Ferwerda G. Bates S. Mora-Montes H. M. . (2007). Immune recognition of *Candida albicans* β-glucan by dectin-1. J. Infect. Dis. 196, 1565–1571. doi: 10.1086/523110 18008237 PMC2655640

[B47] Grahl N. Demers E. G. Lindsay A. K. Harty C. E. Willger S. D. Piispanen A. E. . (2015). Mitochondrial activity and Cyr1 are key regulators of Ras1 activation of *C. albicans* virulence pathways. PloS Pathog. 11, e1005133. doi: 10.1371/journal.ppat.1005133 26317337 PMC4552728

[B48] Guimarães R. Milho C. Liberal Â. Silva J. Fonseca C. Barbosa A. . (2021). Antibiofilm potential of medicinal plants against *Candida* spp. oral biofilms: a review. Antibiotics 10, 1142. doi: 10.3390/antibiotics10091142 34572724 PMC8464735

[B49] Hall R. A. Cottier F. Mühlschlegel F. A. (2009). Molecular networks in the fungal pathogen *Candida albicans*. Adv. Appl. Microbiol. 67, 191–212. doi: 10.1016/S0065-2164(08)01006-X 19245940

[B50] Hameed S. Hans S. Monasky R. Thangamani S. Fatima Z. (2021). Understanding human microbiota offers novel and promising therapeutic options against *Candida* infections. Pathogens 10, 183. doi: 10.3390/pathogens10020183 33572162 PMC7915436

[B51] Han T.-L. Cannon R. D. Villas-Bôas S. G. (2011). The metabolic basis of *Candida albicans* morphogenesis and quorum sensing. Fungal Genet. Biol. 48, 747–763. doi: 10.1016/j.fgb.2011.04.002 21513811

[B52] Hebecker B. Naglik J. R. Hube B. Jacobsen I. D. (2014). Pathogenicity mechanisms and host response during oral *Candida albicans* infections. Expert Rev. Anti-Infective Ther. 12, 867–879. doi: 10.1586/14787210.2014.916210 24803204

[B53] Hernández-Santos N. Gaffen S. L. (2012). Th17 cells in immunity to *Candida albicans*. Cell. Host Microbe 11, 425–435. doi: 10.1016/j.chom.2012.04.008 22607796 PMC3358697

[B54] Hogan D. A. Muhlschlegel F. A. (2011). *Candida albicans* developmental regulation: adenylyl cyclase as a coincidence detector of parallel signals. Curr. Opin. Microbiol. 14, 682–686. doi: 10.1016/j.mib.2011.09.014 22014725

[B55] Hogan D. A. Sundstrom P. (2009). The Ras/cAMP/PKA signaling pathway and virulence in *Candida albicans*. Future Microbiol. 4, 1263–1270. doi: 10.2217/fmb.09.106 19995187

[B56] Hoyer L. L. Green C. B. Oh S.-H. Zhao X. (2008). Discovering the secrets of the *Candida albicans* agglutinin-like sequence (ALS) gene family–a sticky pursuit. Med. Mycol. 46, 1–15. doi: 10.1080/13693780701435317 17852717 PMC2742883

[B57] Hu L. Bai G. Xu Q. Zhao G. Jiang N. Yao H. . (2023). Candidalysin amplifies the immune inflammatory response in *Candida albicans* keratitis through the TREM-1/DAP12 pathway. Int. Immunopharmacol. 119, 110195. doi: 10.1016/j.intimp.2023.110195 37087869

[B58] Hu L. Zhou M. Young A. Zhao W. Yan Z. (2019). *In vivo* effectiveness and safety of probiotics on prophylaxis and treatment of oral candidiasis: a systematic review and meta-analysis. BMC Oral. Health 19, 1–12. doi: 10.1186/s12903-019-0841-2 31291932 PMC6621984

[B59] Huang G. (2012). Regulation of phenotypic transitions in the fungal pathogen *Candida albicans*. Virulence 3, 251–261. doi: 10.4161/viru.20010 22546903 PMC3442837

[B60] Huang H. Harcus D. Whiteway M. (2008). Transcript profiling of a MAP kinase pathway in *C. albicans*. Microbiol. Res. 163, 380–393. doi: 10.1016/j.micres.2008.03.001 18467082

[B61] Huang M.-C. Shen M. Huang Y.-J. Lin H.-C. Chen C.-T. (2018). Photodynamic inactivation potentiates the susceptibility of antifungal agents against the planktonic and biofilm cells of *Candida albicans*. Int. J. Mol. Sci. 19, 434. doi: 10.3390/ijms19020434 29389883 PMC5855656

[B62] Ishikawa K. H. Mayer M. P. Miyazima T. Y. Matsubara V. H. Silva E. G. Paula C. R. . (2015). A multispecies probiotic reduces oral *Candida* colonization in denture wearers. J. Prosthodontics 24, 194–199. doi: 10.1111/jopr.12198 25143068

[B63] Jallow S. Govender N. P. (2021). Ibrexafungerp: a first-in-class oral triterpenoid glucan synthase inhibitor. J. Fungi 7, 163. doi: 10.3390/jof7030163 33668824 PMC7996284

[B64] Javed F. Samaranayake L. P. Romanos G. E. (2014). Treatment of oral fungal infections using antimicrobial photodynamic therapy: a systematic review of currently available evidence. Photochemical Photobiological Sci. 13, 726–734. doi: 10.1039/c3pp50426c 24686309

[B65] Jiang Q. Stamatova I. Kari K. Meurman J. (2015). Inhibitory activity *in vitro* of probiotic lactobacilli against oral *Candida* under different fermentation conditions. Benefic. Microbes 6, 361–368. doi: 10.3920/BM2014.0054 25380800

[B66] Jørgensen M. R. (2024). Pathophysiological microenvironments in oral candidiasis. APMIS 132, 956–973. doi: 10.1111/apm.13412 38571459

[B67] Joshi K. M. Shelar A. Kasabe U. Nikam L. K. Pawar R. A. Sangshetti J. . (2022). Biofilm inhibition in *Candida albicans* with biogenic hierarchical zinc-oxide nanoparticles. Biomater. Adv. 134, 112592. doi: 10.1016/j.msec.2021.112592 35527134

[B68] Kissmann A.-K. Mildenberger V. Krämer M. Alpízar-Pedraza D. Martell-Huguet E. M. Perez-Erviti J. A. . (2025). Anti-biofilm peptides can rescue fluconazole and amphotericin B efficacies against *Candida albicans*. Sci. Rep. 15, 24593. doi: 10.1038/s41598-025-10315-4 40634492 PMC12241370

[B69] Klis F. M. Sosinska G. J. De Groot P. W. Brul S. (2009). Covalently linked cell wall proteins of *Candida albicans* and their role in fitness and virulence. FEMS Yeast Res. 9, 1013–1028. doi: 10.1111/j.1567-1364.2009.00541.x 19624749

[B70] Komalapriya C. Kaloriti D. Tillmann A. T. Yin Z. Herrero-de-Dios C. Jacobsen M. D. . (2015). Integrative model of oxidative stress adaptation in the fungal pathogen *Candida albicans*. PloS One 10, e0137750. doi: 10.1371/journal.pone.0137750 26368573 PMC4569071

[B71] Kraft-Bodi E. Jørgensen M. Keller M. Kragelund C. Twetman S. (2015). Effect of probiotic bacteria on oral *Candida* in frail elderly. J. Dent. Res. 94, 181S–186S. doi: 10.1177/0022034515595950 26202995

[B72] Kullas A. L. Martin S. J. Davis D. (2007). Adaptation to environmental pH: integrating the Rim101 and calcineurin signal transduction pathways. Mol. Microbiol. 66, 858–871. doi: 10.1111/j.1365-2958.2007.05929.x 17927701

[B73] Kumar D. Kumar A. (2024). Molecular determinants involved in *Candida albicans* biofilm formation and regulation. Mol. Biotechnol. 66, 1640–1659. doi: 10.1007/s12033-023-00796-x 37410258

[B74] Lazãr V. Chifiriuc M. C. (2010). Medical significance and new therapeutical strategies for biofilm associated infections. Romanian Arch. Microbiol. Immunol. 69, 125–138. 21434589

[B75] Lenka S. Swain S. K. Bhuyan R. Sahu M. C. (2020). Fungal infection in the oral cavity: A review. Int. J. Curr. Res. Rev. 12, 149–153. doi: 10.31782/IJCRR.2020.121821

[B76] Li H. Zhou X. Huang Y. Liao B. Cheng L. Ren B. (2021). Reactive oxygen species in pathogen clearance: the killing mechanisms, the adaption response, and the side effects. Front. Microbiol. 11, 622534. doi: 10.3389/fmicb.2020.622534 33613470 PMC7889972

[B77] Lionakis M. S. Drummond R. A. Hohl T. M. (2023). Immune responses to human fungal pathogens and therapeutic prospects. Nat. Rev. Immunol. 23, 433–452. doi: 10.1038/s41577-022-00826-w 36600071 PMC9812358

[B78] Liu H. (2001). Transcriptional control of dimorphism in *Candida albicans*. Curr. Opin. Microbiol. 4, 728–735. doi: 10.1016/S1369-5274(01)00275-2 11731326

[B79] Maciel C. M. Piva M. R. Ribeiro M. A. G. de Santana Santos T. Ribeiro C. F. Martins‐Filho P. R. S. (2016). Methylene blue‐mediated photodynamic inactivation followed by low‐laser therapy versus miconazole gel in the treatment of denture stomatitis. J. Prosthodontics 25, 28–32. doi: 10.1111/jopr.12284 26375599

[B80] Mallick P. Sahoo M. K. Debta P. Sahu M. C. (2018). Surveillance of *Candida albicans* and their Antifungal Susceptibility in Oral Candidasis. Indian J. Public Health Res. Dev. 9, 2346–2351. doi: 10.5958/0976-5506.2018.02212.X

[B81] Mallick U. Sahu B. K. Hegde R. Jena P. Turuk J. Sahu M. C. . (2025). Antifungal resistance in vaginal candidiasis among reproductive-age women: A review. Curr. Pharmacutical Biotechnol. 27, 1274–1294. doi: 10.2174/0113892010368329250503175104 40353419

[B82] Mallick U. Sahu B. K. Patra S. K. Sahu M. C. Panda S. K. (2026). Breaking the barrier: cutting‐Edge microbial strategies against *candida* biofilm infections. APMIS 134, e70214. doi: 10.1111/apm.70214 42047000

[B83] Mathew D. Thomas B. Sudheep N. Nair S. Radhakrishnan E. (2025). Mangifera indica phytochemical enriched ZnO-Amphotericin B Nanoconjugates: Enhanced hydrophilicity, reduced toxicity and pH-sensitive release for targeted anti-*Candida* therapy. Adv. Powder Technol. 36, 104895. doi: 10.1016/j.apt.2025.10489542574925

[B84] Matsubara V. H. Wang Y. Bandara H. Mayer M. P. A. Samaranayake L. P. (2016). Probiotic lactobacilli inhibit early stages of *Candida albicans* biofilm development by reducing their growth, cell adhesion, and filamentation. Appl. Microbiol. Biotechnol. 100, 6415–6426. doi: 10.1007/s00253-016-7527-3 27087525

[B85] Mayer F. L. Wilson D. Hube B. (2013). *Candida albicans* pathogenicity mechanisms. Virulence 4, 119–128. doi: 10.4161/viru.22913 23302789 PMC3654610

[B86] McDonough K. A. Rodriguez A. (2012). The myriad roles of cyclic AMP in microbial pathogens: from signal to sword. Nat. Rev. Microbiol. 10, 27–38. doi: 10.1038/nrmicro2688 22080930 PMC3785115

[B87] Mima E. Vergani C. E. MaChado A. L. Massucato E. M. S. Colombo A. L. Bagnato V. S. . (2012). Comparison of photodynamic therapy versus conventional antifungal therapy for the treatment of denture stomatitis: a randomized clinical trial. Clin. Microbiol. Infection 18, E380–E388. doi: 10.1111/j.1469-0691.2012.03933.x 22731617

[B88] Mitchell K. Taff H. Cuevas M. Reinicke E. Sanchez H. Andes D. (2013). Role of matrix β-1, 3 glucan in antifungal resistance of non-albicans *Candida* biofilms. Antimicrob. Agents Chemother. 57, 1918–1920. doi: 10.1128/aac.02378-12 23318790 PMC3623361

[B89] Miyazima T. Y. Ishikawa K. H. Mayer M. P. A. Saad S. M. I. Nakamae A. E. M. (2017). Cheese supplemented with probiotics reduced the *Candida* levels in denture wearers—RCT. Oral. Dis. 23, 919–925. doi: 10.1111/odi.12669 28346730

[B90] Modrzewska B. Kurnatowski P. (2015). Adherence of *Candida* sp. to host tissues and cells as one of its pathogenicity features. Ann. Parasitol. 61, 3–9. 25911031

[B91] Molaeitabari A. Dahms T. E. (2025). Blocking the shikimate pathway amplifies the impact of carvacrol on biofilm formation in *Candida albicans*. Microbiol. Spectr. 13, e02754-02724. doi: 10.1128/spectrum.02754-24 39918333 PMC11878086

[B92] Monteiro D. Arias L. Fernandes R. Deszo da Silva L. De Castilho M. da Rosa T. . (2017). Antifungal activity of tyrosol and farnesol used in combination against *Candida* species in the planktonic state or forming biofilms. J. Appl. Microbiol. 123, 392–400. doi: 10.1111/jam.13513 28622460

[B93] Morici P. Fais R. Rizzato C. Tavanti A. Lupetti A. (2016). Inhibition of *Candida albicans* biofilm formation by the synthetic lactoferricin derived peptide hLF1-11. PloS One 11, e0167470. doi: 10.1371/journal.pone.0167470 27902776 PMC5130267

[B94] Mundula T. Ricci F. Barbetta B. Baccini M. Amedei A. (2019). Effect of probiotics on oral candidiasis: a systematic review and meta-analysis. Nutrients 11, 2449. doi: 10.3390/nu11102449 31615039 PMC6836010

[B95] Naglik J. R. Challacombe S. J. Hube B. (2003a). *Candida albicans* secreted aspartyl proteinases in virulence and pathogenesis. Microbiol. Mol. Biol. Rev. 67, 400–428. doi: 10.1128/mmbr.67.3.400-428.2003 12966142 PMC193873

[B96] Naglik J. R. Gaffen S. L. Hube B. (2019). Candidalysin: discovery and function in *Candida albicans* infections. Curr. Opin. Microbiol. 52, 100–109. doi: 10.1016/j.mib.2019.06.002 31288097 PMC6687503

[B97] Naglik J. R. Rodgers C. A. Shirlaw P. J. Dobbie J. L. Fernandes-Naglik L. L. Greenspan D. . (2003b). Differential expression of *Candida albicans* secreted aspartyl proteinase and phospholipase B genes in humans correlates with active oral and vaginal infections. J. Infect. Dis. 188, 469–479. doi: 10.1086/376536 12870130

[B98] Nazzaro F. Fratianni F. Coppola R. De Feo V. (2017). Essential oils and antifungal activity. Pharmaceuticals 10, 86. doi: 10.3390/ph10040086 29099084 PMC5748643

[B99] Nett J. E. Andes D. R. (2020). Contributions of the biofilm matrix to *Candida* pathogenesis. J. Fungi 6, 21. doi: 10.3390/jof6010021 32028622 PMC7151000

[B100] Nett J. E. Sanchez H. Cain M. T. Andes D. R. (2010). Genetic basis of *Candida* biofilm resistance due to drug-sequestering matrix glucan. J. Infect. Dis. 202, 171–175. doi: 10.1086/651200 20497051 PMC2880631

[B101] Nikou S.-A. Kichik N. Brown R. Ponde N. O. Ho J. Naglik J. R. . (2019). *Candida albicans* interactions with mucosal surfaces during health and disease. Pathogens 8, 53. doi: 10.3390/pathogens8020053 31013590 PMC6631630

[B102] Nuncã A.-M. Coculescu E. C. Coculescu B.-I. (2022). The role of general factors in the pathogenesis of oral candidiasis. Romanian Arch. Microbiol. Immunol. 81, 271–277. doi: 10.54044/RAMI.2022.04.04

[B103] Nunes I. P. Crugeira P. J. Sampaio F. J. de Oliveira S. C. Azevedo J. M. Santos C. L. . (2023). Evaluation of dual application of photodynamic therapy—PDT in *Candida albicans*. Photodiagn. Photodyn. Ther. 42, 103327. doi: 10.1016/j.pdpdt.2023.103327 36773756

[B104] Parker R. A. Gabriel K. T. Graham K. D. Butts B. K. Cornelison C. T. (2022). Antifungal activity of select essential oils against *Candida auris* and their interactions with antifungal drugs. Pathogens 11, 821. doi: 10.3390/pathogens11080821 35894044 PMC9331469

[B105] Patel M. (2022). Oral cavity and *Candida albicans*: Colonisation to the development of infection. Pathogens 11, 335. doi: 10.3390/pathogens11030335 35335659 PMC8953496

[B106] Pellissari C. V. G. Jorge J. H. Marin L. M. Sabino-Silva R. Siqueira W. L. (2021). Statherin-derived peptides as antifungal strategy against *Candida albicans*. Arch. Oral. Biol. 125, 105106. doi: 10.1016/j.archoralbio.2021.105106 33740624

[B107] Pfaller M. A. Diekema D. J. Turnidge J. D. Castanheira M. Jones R. N. (2019). Twenty years of the SENTRY antifungal surveillance program: results for *Candida* species from 1997–2016. Oxford Univ. Press U.S. 6, S79–S94. doi: 10.1093/ofid/ofy358 30895218 PMC6419901

[B108] Pflipsen M. Zenchenko Y. (2017). Nutrition for oral health and oral manifestations of poor nutrition and unhealthy habits. Gen. Dent. 65, 36–43. 29099364

[B109] Polke M. Leonhardt I. Kurzai O. Jacobsen I. D. (2018). Farnesol signalling in *Candida albicans*–more than just communication. Crit. Rev. Microbiol. 44, 230–243. doi: 10.1080/1040841X.2017.1337711 28609183

[B110] Priya A. Pandian S. K. (2022). Biofilm and hyphal inhibitory synergistic effects of phytoactives piperine and cinnamaldehyde against *Candida albicans*. Med. Mycol. 60, myac039. doi: 10.1093/mmy/myac039 35661216

[B111] Quindós G. Gil-Alonso S. Marcos-Arias C. Sevillano E. Mateo E. Jauregizar N. . (2019). Therapeutic tools for oral candidiasis: Current and new antifungal drugs. Med. Oral Patologia Oral. Y. Cirugia Bucal 24, e172. doi: 10.4317/medoral.22978 30818309 PMC6441600

[B112] Rajão A. Silva J. P. Almeida-Nunes D. L. Rompante P. Rodrigues C. F. Andrade J. C. (2025). Limosilactobacillus reuteri AJCR4: A potential probiotic in the fight against oral *Candida* spp. biofilms. Int. J. Mol. Sci. 26, 638. doi: 10.3390/ijms26020638 39859352 PMC11766303

[B113] Ramage G. Saville S. P. Thomas D. P. Lopez-Ribot J. L. (2005). *Candida* biofilms: an update. Eukaryotic Cell. 4, 633–638. doi: 10.1128/ec.4.4.633-638.2005 15821123 PMC1087806

[B114] Ramírez-Zavala B. Weyler M. Gildor T. Schmauch C. Kornitzer D. Arkowitz R. . (2013). Activation of the Cph1-dependent MAP kinase signaling pathway induces white-opaque switching in *Candida albicans*. PloS Pathog. 9, e1003696. doi: 10.1371/journal.ppat.1003696 24130492 PMC3795047

[B115] Rao H. Choo S. Rajeswari Mahalingam S. R. Adisuri D. S. Madhavan P. Md. Akim A. . (2021). Approaches for mitigating microbial biofilm-related drug resistance: a focus on micro-and nanotechnologies. Molecules 26, 1870. doi: 10.3390/molecules26071870 33810292 PMC8036581

[B116] Rath S. Behera I. C. Sahu M. C. (2018). Antifungal susceptibility of *Candida* species isolated from urine from patients in a neurosciences intensive care unit. Apollo Med. 15, 219–222. doi: 10.4103/am.am_43_18

[B117] Raught B. Gingras A.-C. Sonenberg N. (2001). The target of rapamycin (TOR) proteins. Proc. Natl. Acad. Sci. 98, 7037–7044. doi: 10.1073/pnas.121145898 11416184 PMC34619

[B118] Riekhof W. R. Nickerson K. (2017). Quorum sensing in *Candida albicans*: farnesol versus farnesoic acid. FEBS Lett. 591, 1637–1640. doi: 10.1002/1873-3468.12636 28603849

[B119] Rodrigues C. F. Rodrigues M. E. Henriques M. (2019). *Candida* sp. infections in patients with diabetes mellitus. J. Clin. Med. 8, 76. doi: 10.3390/jcm8010076 30634716 PMC6352194

[B120] Rodríguez-Arias R. J. Guachi-Álvarez B. O. Montalvo-Vivero D. E. MaChado A. (2022). Lactobacilli displacement and *Candida albicans* inhibition on initial adhesion assays: a probiotic analysis. BMC Res. Notes 15, 239. doi: 10.1186/s13104-022-06114-z 35799214 PMC9264498

[B121] Sahu B. K. Panda S. K. Mallick U. Panda S. H. Sahu M. C. (2026a). The role of probiotics in restoring and maintaining vaginal microbiome health: a review. Infect. Immun. 94, e00011-00026. doi: 10.1128/iai.00011-26 42360164 PMC13460080

[B122] Sahu B. K. Patra S. K. Sahu M. C. Panda S. K. (2026b). Natural product based approaches to overcome *Candida glabrata* and emerging AMR threats. Front. Antibiot. 5, 1767032. doi: 10.3389/frabi.2026.1767032 41743597 PMC12930641

[B123] Sahu B. K. Sahu M. C. Panda S. K. (2026c). Phytochemicals as novel antifungal agents against *Candida* species. Curr. Pharm. Biotechnol. doi: 10.2174/0113892010500244260721094728 42522315

[B124] Santos A. Oliveira-Filho A. Teixeira B. Borchardt H. Galvão J. Medeiros M. . (2024). Lavandula dentata L. essential oil: a promising antifungal and antibiofilm agent against oral *Candida albicans*. Braz. J. Biol. 84, e283646. doi: 10.1590/1519-6984.283646 39109727

[B125] Sardi J. Scorzoni L. Bernardi T. Fusco-Almeida A. Mendes Giannini M. J. S. (2013). *Candida* species: current epidemiology, pathogenicity, biofilm formation, natural antifungal products and new therapeutic options. J. Med. Microbiol. 62, 10–24. doi: 10.1099/jmm.0.045054-0 23180477

[B126] Sawant B. Khan T. (2017). Recent advances in delivery of antifungal agents for therapeutic management of candidiasis. Biomedicine Pharmacotherapy 96, 1478–1490. doi: 10.1016/j.biopha.2017.11.127 29223551

[B127] Scwingel A. R. Barcessat A. R. P. Núñez S. C. Ribeiro M. S. (2012). Antimicrobial photodynamic therapy in the treatment of oral candidiasis in HIV-infected patients. Photomed. Laser Surg. 30, 429–432. doi: 10.1089/pho.2012.3225 22730912

[B128] Seneviratne C. Jin L. Samaranayake L. (2008). Biofilm lifestyle of *Candida*: a mini review. Oral. Dis. 14, 582–590. doi: 10.1111/j.1601-0825.2007.01424.x 19076549

[B129] Shahabudin S. Azmi N. S. Lani M. N. Mukhtar M. Hossain M. S. (2024). *Candida albicans* skin infection in diabetic patients: An updated review of pathogenesis and management. Mycoses 67, e13753. doi: 10.1111/myc.13753 38877612

[B130] Shankar Raut J. Mohan Karuppayil S. (2016). Phytochemicals as inhibitors of *Candida* biofilm. Curr. Pharm. Des. 22, 4111–4134. doi: 10.1111/myc.13753 27262331

[B131] Shariati A. Didehdar M. Razavi S. Heidary M. Soroush F. Chegini Z. (2022). Natural compounds: a hopeful promise as an antibiofilm agent against *Candida* species. Front. Pharmacol. 13, 917787. doi: 10.3389/fphar.2022.917787 35899117 PMC9309813

[B132] Sharma A. (2019). Oral candidiasis: An opportunistic infection: A review. Int. J. Appl. Dental Sci. 5, 23–27.

[B133] Sharma R. K. Ghose R. (2015). Synthesis of zinc oxide nanoparticles by homogeneous precipitation method and its application in antifungal activity against *Candida albicans*. Ceram. Int. 41, 967–975. doi: 10.1016/j.ceramint.2014.09.01642574925

[B134] Sharma A. Singhai H. Rathee S. Soni S. Patil U. K. (2026). Targeting *Candida albicans* infections through synergistic phytochemical and antifungal combinations. Curr. Pharm. Des. doi: 10.2174/0113816128499052260715140756 42515907

[B135] Shaw K. J. Ibrahim A. S. (2020). Fosmanogepix: a review of the first-in-class broad spectrum agent for the treatment of invasive fungal infections. J. Fungi 6, 239. doi: 10.3390/jof6040239 33105672 PMC7711534

[B136] Shimasawa M. Sakamaki J. Maeda T. Mizushima N. (2023). The pH-sensing Rim101 pathway regulates cell size in budding yeast. J. Biol. Chem. 299, 102973. doi: 10.1016/j.jbc.2023.102973 36738789 PMC10011510

[B137] Singh S. Kumari S. (2024). To investigate the clinical presentation, diagnosis, and management of oral candidiasis in immunocompetent patients. Int. J. Acad. Med. Pharm. 6, 837–842. doi: 10.47009/jamp.2024.6.4.164

[B138] Song Y.-G. Lee S.-H. (2017). Inhibitory effects of *Lactobacillus rhamnosus* and *Lactobacillus casei* on *Candida* biofilm of denture surface. Arch. Oral. Biol. 76, 1–6. doi: 10.1016/j.archoralbio.2016.12.014 28063305

[B139] Song P. Peng G. Yue H. Ogawa T. Ikeda S. Okumura K. . (2022). Candidalysin, a virulence factor of *Candida albicans*, stimulates mast cells by mediating cross-talk between signaling pathways activated by the dectin-1 receptor and MAPKs. J. Clin.Immunol. 42, 1009–1025. doi: 10.1007/s10875-022-01267-9 35420364

[B140] Sudbery P. E. (2011). Growth of *Candida albicans* hyphae. Nat. Rev. Microbiol. 9, 737–748. doi: 10.1038/nrmicro2636 21844880

[B141] Sun C. Zhao X. Jiao Z. Peng J. Zhou L. Yang L. . (2022). The antimicrobial peptide AMP-17 derived from Musca domestica inhibits biofilm formation and eradicates mature biofilm in *Candida albicans*. Antibiotics 11, 1474. doi: 10.3390/antibiotics11111474 36358129 PMC9686669

[B142] Thakare R. Dasgupta A. Chopra S. (2019). Ibrexafungerp. 1, 3-Beta-Glucan synthase inhibitor, triterpenoid antifungal agent. Drugs Future 44, 256–275. doi: 10.1358/dof.2019.44.4.296284442174228

[B143] Thakare R. Dasgupta A. Chopra S. (2021). Fosmanogepix. GPI-anchored wall transfer protein 1 (Gwt1)(fungal) inhibitor, treatment of invasive fungal infections. Drugs Future 46, 253–271. doi: 10.1358/dof.2021.46.4.322338442174228

[B144] Tian X. Ding H. Ke W. Wang L. (2021). Quorum sensing in fungal species. Annu. Rev. Microbiol. 75, 449–469. doi: 10.1146/annurev-micro-060321-045510 34348030

[B145] Tong Y. Zhang J. Wang L. Wang Q. Huang H. Chen X. . (2021). Hyper-synergistic antifungal activity of rapamycin and peptide-like compounds against *Candida albicans* orthogonally via Tor1 kinase. ACS Infect. Dis. 7, 2826–2835. doi: 10.1021/acsinfecdis.1c00448 34514778

[B146] Tsui C. Kong E. F. Jabra-Rizk M. A. (2016). Pathogenesis of Candida albicans biofilm. FEMS Pathog. Dis. 74, ftw018. doi: 10.1093/femspd/ftw018 26960943 PMC5975230

[B147] Tuan D. A. Masak J. (2026). Natural-compound adjuvants dismantle Candida biofilms: mechanisms, design rules, and biofilm-aware pharmacology. Curr. Microbiol. 83, 131. doi: 10.1007/s00284-025-04713-0 41524898 PMC12795885

[B148] Tullio V. Roana J. Scalas D. Mandras N. (2019). Evaluation of the antifungal activity of Mentha x piperita (Lamiaceae) of Pancalieri (Turin, Italy) essential oil and its synergistic interaction with azoles. Molecules 24, 3148. doi: 10.3390/molecules24173148 31470602 PMC6749244

[B149] Vila T. Sultan A. S. Montelongo-Jauregui D. Jabra-Rizk M. A. (2020). Oral candidiasis: A disease of opportunity. J. Fungi 6, 15. doi: 10.3390/jof6010015 31963180 PMC7151112

[B150] Weisman R. (2016). Target of rapamycin (TOR) regulates growth in response to nutritional signals. Microbiol. Spectr. 4, 1–13. doi: 10.1128/microbiolspec.funk-0006-2016 27763256

[B151] Wiederhold N. P. (2018). The antifungal arsenal: alternative drugs and future targets. Int. J. Antimicrob. Agents 51, 333–339. doi: 10.1016/j.ijantimicag.2017.09.002 28890395

[B152] Williams D. Lewis M. (2011). Pathogenesis and treatment of oral candidosis. J. Oral. Microbiol. 3, 5771. doi: 10.3402/jom.v3i0.5771 21547018 PMC3087208

[B153] Yamanaka-Takaichi M. Ghanian S. Katzka D. A. Torgerson R. R. Alavi A. (2022). *Candida* infection associated with anti-IL-17 medication: A systematic analysis and review of the literature: M. Yamanaka-Takaichi et al. Am. J. Clin. Dermatol. 23, 469–480. doi: 10.1007/s40257-022-00686-z 35428934

[B154] Yan H. Fang T. Xu H. Jiang L. (2020). The pH-sensing Rim101 pathway positively regulates the transcriptional expression of the calcium pump gene PMR1 to affect calcium sensitivity in budding yeast. Biochem. Biophys. Res. Commun. 532, 453–458. doi: 10.1016/j.bbrc.2020.08.083 32891431

[B155] Yassin M. T. Al-Otibi F. O. Maniah K. Mohamed S. Hasan K. A. Negi A. . (2025). Synergistic antifungal efficiency of eco-friendly synthesized zinc oxide nanoparticles in combination with fluconazole against drug-resistant candidal strains. Pol. J. Environ. Stud. 34, 1851–1865. doi: 10.15244/pjoes/18814422837105

[B156] Yassin M. T. Elgorban A. M. Al-Askar A. A. Sholkamy E. N. Ameen F. Maniah K. (2023). Synergistic anticandidal activities of greenly synthesized ZnO nanomaterials with commercial antifungal agents against candidal infections. Micromachines 14, 209. doi: 10.3390/mi14010209 36677271 PMC9865458

[B157] Zahraoui E. M. (2025). Essential oils: Antifungal activity and study methods. Moroccan J. Agric. Sci. 6, 99–108. doi: 10.5281/zenodo.1547243742270437

[B158] Zangl I. Pap I.-J. Aspöck C. Schüller C. (2019). The role of *Lactobacillus* species in the control of *Candida* via biotrophic interactions. Microbial Cell. 7, 1. doi: 10.15698/mic2020.01.702 31921929 PMC6946018

[B159] Zeise K. D. Woods R. J. Huffnagle G. B. (2021). Interplay between *Candida albicans* and lactic acid bacteria in the gastrointestinal tract: Impact on colonization resistance, microbial carriage, opportunistic infection, and host immunity. Clin. Microbiol. Rev. 34, e00323-00320. doi: 10.1128/CMR.00323-20 34259567 PMC8404691

